# Genetic determinants of inherited susceptibility to hypercholesterolemia – a comprehensive literature review

**DOI:** 10.1186/s12944-017-0488-4

**Published:** 2017-06-02

**Authors:** CS Paththinige, ND Sirisena, VHW Dissanayake

**Affiliations:** Human Genetics Unit, Faculty of Medicine, University of Colombo, Kynsey Road, Colombo, 00800 Sri Lanka

**Keywords:** Hypercholesterolemia, Lipid metabolism, Single nucleotide variants, Genome-wide association studies, Candidate gene studies

## Abstract

Hypercholesterolemia is a strong determinant of mortality and morbidity associated with cardiovascular diseases and a major contributor to the global disease burden. Mutations in four genes (*LDLR*, *APOB*, *PCSK9* and *LDLRAP1*) account for the majority of cases with familial hypercholesterolemia. However, a substantial proportion of adults with hypercholesterolemia do not have a mutation in any of these four genes. This indicates the probability of having other genes with a causative or contributory role in the pathogenesis of hypercholesterolemia and suggests a polygenic inheritance of this condition. Here in, we review the recent evidence of association of the genetic variants with hypercholesterolemia and the three lipid traits; total cholesterol (TC), HDL-cholesterol (HDL-C) and LDL-cholesterol (LDL-C), their biological pathways and the associated pathogenetic mechanisms. Nearly 80 genes involved in lipid metabolism (encoding structural components of lipoproteins, lipoprotein receptors and related proteins, enzymes, lipid transporters, lipid transfer proteins, and activators or inhibitors of protein function and gene transcription) with single nucleotide variants (SNVs) that are recognized to be associated with hypercholesterolemia and serum lipid traits in genome-wide association studies and candidate gene studies were identified. In addition, genome-wide association studies in different populations have identified SNVs associated with TC, HDL-C and LDL-C in nearly 120 genes within or in the vicinity of the genes that are not known to be involved in lipid metabolism. Over 90% of the SNVs in both these groups are located outside the coding regions of the genes. These findings indicates that there might be a considerable number of unrecognized processes and mechanisms of lipid homeostasis, which when disrupted, would lead to hypercholesterolemia. Knowledge of these molecular pathways will enable the discovery of novel treatment and preventive methods as well as identify the biochemical and molecular markers for the risk prediction and early detection of this common, yet potentially debilitating condition.

**Electronic supplementary material:**

The online version of this article (doi:10.1186/s12944-017-0488-4) contains supplementary material, which is available to authorized users.

## Background

Hypercholesterolemia is a common disorder which was reported in 39% of the worlds’ adult population in 2008 [1]. The association between hypercholesterolemia, more specifically elevated low density lipoprotein-cholesterol (LDL-C) levels, and coronary heart disease is already recognized by the evidence from many prospective epidemiological studies [2]. Although the association between the hypercholesterolemia and stroke is not strongly established, clinical trials and meta-analysis of the effect of statins have demonstrated a reduction in stroke risk following treatment with statins, suggesting an association between hypercholesterolemia and the risk of stroke [3]. Coronary heart disease and stroke are the two leading causes of death worldwide and by the year 2030, they are projected to account for nearly 25% of deaths [4, 5]. Association of hypercholesterolemia with these two conditions makes it a major contributor to the global disease burden and cardiovascular disease associated mortality and morbidity.

**Fig. 1:**
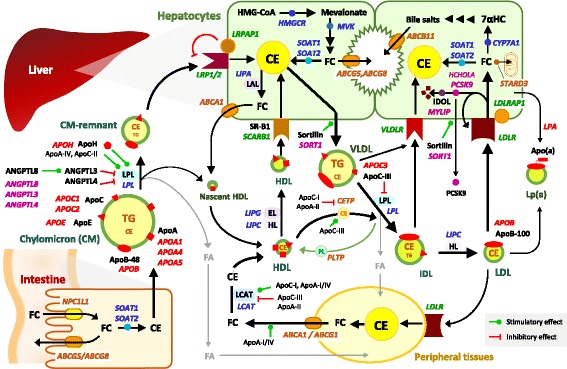
Schematic presentation of the pathways of cholesterol/lipoprotein metabolism and the genes involved. 7α-hydroxy cholesterol: 7α HC, Angiopoietin-like proteins: ANGPTL, Apolipoproteins: Apo., Cholesterol esters: CE, Cholesteryl ester transfer protein: CETP, Chylomicrons: CM, Endothelial lipase: EL, Fatty acids: FA, Free cholesterol: FC, Hepatic lipase: HL, High density lipoprotein: HDL, Inducible degrader of LDL receptor: IDOL, Intermediate-density lipoproteins: IDL, Lecithin: cholesterolacyl transferase: LCAT, Lipoprotein lipase: LPL, Lipoprotein-a: Lp(a), Low density lipoprotein: LDL, Lysosomal acid lipase: LAL, Scavenger receptor B1: SR-B1, Triglycerides: TG, Very low density lipoprotein: VLDL. Genes encoding lipoprotein receptors and receptor-related/associated proteins are in green colour. Genes encoding proteins with an enzymatic function in lipid/lipoprotein metabolism are in blue colour. Genes encoding lipid transporters and lipid transfer proteins are in brown colour. Genes encoding proteins with a regulatory function in lipid homeostasis are in purple colour

Hypercholesterolemia corresponds with an elevated plasma LDL-C level because LDL is the main carrier of cholesterol in plasma. Dietary cholesterol absorbed by the intestine is initially packed into chylomicrons. Chylomicrons are broken down by lipoprotein lipase (LPL) and the fatty acids are transported into the muscles and adipose tissue, while the chylomicron remnants enter the liver. In the liver, very-low-density lipoproteins (VLDL) are produced and secreted into the bloodstream. VLDL is metabolized by LPL to produce intermediate-density lipoproteins (IDL) which are then converted in to LDL. Nascent high density lipoprotein (HDL) particles are also produced by the liver, which converts to HDL by incorporating cholesteryl esters derived from cholesterol liberated from peripheral tissues. Thereby, HDL is involved in reverse cholesterol transport from the peripheral tissues to the liver. Cholesteryl esters carried in HDL particles are transferred to LDLs by the action of cholesteryl ester transfer protein (CETP). LDL particles carry these cholesteryl esters and are taken up by the liver and to a lesser extent by peripheral tissues. The uptake and degradation of LDL in the liver depends on the binding of LDL particles to the cell surface receptors (LDL-receptors) of the hepatocytes. The process of LDL uptake and degradation is explained in 4 steps. The LDL receptor protein is produced in the endoplasmic reticulum of the hepatocytes and its maturation occurs within the Golgi apparatus, then the receptors get expressed on the cell surface. This receptor specifically binds to apolipoprotein B (apoB) in the LDL particle. Then the LDL:LDL-receptor complex is internalized by endocytosis. This internalization process is mediated by the LDL receptor adaptor protein. The receptor molecule is then recycled and the LDL particle undergoes lysosomal degradation. Proprotein convertase subtilisin/kexin type 9 (PCSK9), a protein expressed in the hepatocytes is thought to be involved in the catabolism of LDL-receptors [6] (Fig. 1).

## Monogenic familial hypercholesterolemia vs. polygenic hypercholesterolemia

Mutations of the genes that encode the proteins involved in LDL uptake and catabolism (i.e. LDL-receptor by *LDLR* gene, apolipoprotein-B (ApoB) by *APOB* gene, LDL receptor adaptor protein by *LDLRAP1* gene and PCSK9 protein by *PCSK9* gene) are well-known to cause familial hypercholesterolemia by defective LDL uptake and degradation, which in-turn leads to an elevation of plasma LDL-C level, producing the hypercholesterolemia phenotype. These conditions are characterized by extreme hypercholesterolemia with severe elevation of LDL-C level leading to atherosclerosis and cardiovascular diseases at an early age. They are classically described as monogenic disorders with Mendelian inheritance. Majority of the patients with familial hypercholesterolemia (FH) have a mutation in the *LDLR* gene which is dominantly inherited. Mutations in the *APOB* and *PCSK9* genes accounts for a smaller percentage of autosomal dominant FH. A rare autosomal recessive form of familial hypercholesterolemia is produced by homozygous and compound heterozygous mutations of *LDLRAP1* gene [6, 7].

Recently, novel loci for autosomal dominant FH were mapped to *HCHOLA4* gene at 16q22.1 in a French family [8], and *STAP1* gene at 4q13.2 in a Dutch family [9], using linkage studies and sequencing technology. The function of these genes in lipid metabolism and the association of single nucleotide variants (SNVs) in these loci with serum lipid traits, are not yet clearly identified. However, it was postulated that *HCHOLA4* is involved in intracellular trafficking and degradation of LDL receptors in which the *PCSK9* gene is also involved [8].

It has been reported that nearly 15% of patients with autosomal dominant FH are negative for a mutation in any of the three identified genes described above (i.e. *LDLR, APOB, PCSK*) indicating the probability of having other genes with a causative or contributory role in the pathogenesis of hypercholesterolemia [10]. This suggests a polygenic inheritance of hypercholesterolemia in these patients [11]. Hypercholesterolemia in adults is a complex trait produced by the interplay between the susceptible genotype and the provoking environmental factors such as excessive amount of saturated fat in diet, obesity and physical inactivity. The genetic susceptibility is supposed to be the cumulative effect of the mutations or polymorphisms of the genes with smaller-effect with minor LDL-C raising properties. Interaction among these genes, and between the genes and environment, and the epigenetic mechanisms regulating the gene expression might contribute to the hypercholesterolemia phenotype. Even within the known FH causing genes, some of the variants would be located outside the areas of analysis in the routine sequencing techniques such as those in intronic regions. This highlights the need for a comprehensive gene panel for the evaluation of inherited susceptibility to hypercholesterolemia.

In this review, we explored the recent evidence of association of the genetic variants with hypercholesterolemia and the 3 lipid traits; total cholesterol (TC), HDL-cholesterol (HDL-C) and LDL-C and the biological effects of these genes in lipid homeostasis. The focus is mainly on the genes with a recognized or a potential role in lipid metabolism for which the association with the serum lipid traits; TC, LDL-C and HDL-C was observed in candidate gene association studies, genome-wide association studies (GWAS), genetic linkage studies and other studies with a case–control design. The genes without a recognized role in lipid metabolism, but have been shown to be associated with lipid levels in GWAS are listed in the latter part of this review. The genes associated with plasma triglyceride levels were not included since hypertriglyceridemia is described as a separate clinical entity. The potentially relevant papers during the last 10 years since January 2007 were searched in PubMed using the search terms ‘hypercholesterolemia/genetics’, ‘candidate gene association study’, ‘genome-wide association study’, “genetic linkage study”, “case–control genetic study”. The search was done during January 2017. All the papers cited are full text articles published in English. However, the reference articles cited in this review were selected using the specific criteria mentioned above and for the purpose of highlighting the specific points discussed in this review, and as such the list may not be comprehensive.

## Genetic basis of polygenic hypercholesterolemia – The genes associated with lipid traits; LDL-C, HDL-C and total cholesterol

Recent genetic studies in human subjects including GWAS in many different populations have identified a number of genes or loci that influence the serum lipid levels. Some of these genes have a well-recognized role in lipid metabolism. These include the genes encoding apolipoproteins, lipoprotein receptors and receptor related proteins, lipid transporters and lipid transfer proteins, the enzymes and proteins with a regulatory role in lipoprotein metabolism as well as the proteins that regulate the expression of those genes.

### Genes encoding apolipoproteins

Apolipoproteins are important components of the lipoprotein particles and some act as ligands for receptors for lipoprotein receptors. Some of them play important roles as cofactors, activators or inhibitors of enzymes in lipid metabolism (Fig. 1). Several different classes of apolipoproteins are described and the genes encoding these proteins are observed to be located in clusters.

#### APOB

The link between mutations in *APOB* gene and autosomal dominant FH has already been well-established. The *APOB* gene mapped to chromosome 2p24.1 encodes two isoforms of apolipoprotein-B; apoB-48 and apoB-100. ApoB-100 is synthesized in the liver and is the only type of apolipoprotein of LDL. ApoB-100 maintains the structural integrity of the LDL particle and allows the binding of LDL to LDL-receptor [12]. Few mutations in the *APOB* gene causing defective binding of LDL to LDL-receptor and causing FH have been described, and p.3527 (earlier reported as p.3500) was described as the ‘mutation hotspot’ of *APOB* gene because genetic mutations in majority of patients with FH due to defective apoB were observed at this location [13]. Multiple SNVs associated with serum lipid traits, frequently the LDL-C level were recognized by many GWAS [14–26], and the association of some of these variants (rs693, rs562338, rs506585, rs515135, rs1367117, rs7575840) were replicated in more than one study. In addition, candidate gene association studies have identified some other SNVs and also replicated the association of the SNVs (e.g. rs693, rs1367117) identified in GWAS with the LDL-C levels [27–30].

Mutations in other genes encoding various types of apolipoproteins were identified as the cause for several different types of monogenic dyslipidemias [31]. Several large scale GWAS and meta-analyses for hypercholesterolemia and different lipid traits and many candidate gene association studies have also identified the association between these genes and the hypercholesterolemia phenotype.

#### APOE, APOC1, APOC2, APOC4

These 4 genes are in a cluster mapped to the cytogenetic locus 19q13.32. Apolipoprotein E (ApoE) is the major apolipoprotein of triglyceride rich lipoproteins such as chylomicrons and VLDL. ApoE mediates the catabolism of these lipoproteins by binding to its receptors in the liver and peripheral tissues [32]. A mutations in *APOE* gene (p.Leu167del) causing impaired clearance of chylomicrons and VLDL, and subsequent hypercholesterolemia were observed in two families with French and Italian ancestry [33, 34]. *APOC1*, a gene expressed mainly in the liver encodes apolipoprotein C-I (apoC-I) which plays a key role in HDL and VLDL metabolism by inhibiting the apoE mediated binding of lipoproteins to the receptors. In addition apoC-I acts as an inhibitor of hepatic lipase (HL) and an activator of lecithin: cholesterolacyl transferase (LCAT); enzymes involved in lipid metabolism [32]. Functional studies have also demonstrated an inhibitory effect of this protein on CETP, thereby affecting the lipoprotein level [35]. Apolipoprotein C-II (apoC-II) is a component of VLDL and consists of LPL binding site, to which LPL enzyme binds and gets activated [32]. Apolipoprotein C-IV (apoC-IV) is primarily associated with VLDL and is also assumed to be involved in lipid metabolism [36]. Over the last decade, many GWAS have identified association of the variants at this locus containing *APOE*-*APOC* gene cluster and *TOMM40* gene with plasma lipid traits, especially LDL-C levels [14–26, 37–39]. Most of these studies have identified the association of a common polymorphism; rs4420638 with the serum LDL-C level, which was also replicated in candidate gene studies [27, 29]. Other variants at this locus with replicated evidence of association with LDL-C level in GWAS and candidate gene studies include rs2075650 [30, 38] and rs7412 [38, 39].

#### APOA1, APOA4, APOA5, APOC3

The apolipoprotein gene cluster in 11q23 region consists of *APOA1*, *APOA4*, *APOA5* and *APOC3* genes. Apolipoprotein A-I (apoA-I) is a main structural component of HDL which is important for reverse cholesterol transport from peripheral tissues to the liver for excretion. ApoA-I promotes the cellular efflux of cholesterol, prevents the protease-mediated degradation of ATP binding cassette protein A1 (ABCA1); a transporter protein involved in cellular cholesterol efflux and also acts as a cofactor for LCAT enzyme, all important functions in reverse cholesterol transport [32, 40, 41]. It has been shown that apolipoprotein A-IV (apoA-IV) also has similar effects on reverse cholesterol transport by activating LCAT, stimulating ABCA1 mediated cholesterol efflux, and modulating CETP activity. ApoA-IV also has a stimulating effect on LPL activity [42]. Apolipoprotein A-V (apoA-V), also associated with HDL has been shown to be an important regulator of the plasma triglyceride levels as observed in candidate gene association studies on *APOA5* [43, 44]. However the molecular mechanism of apoA-V activity on lipid homeostasis is not well-recognized and there is conflicting evidence regarding the effect of apoA-V on hepatic VLDL secretion and LPL activity [45]. Apolipoprotein C3 (apoC-III) is closely linked to *APOA* gene cluster at 11q23. ApoC-III impairs the lipoprotein catabolism by hindering the interaction between these lipoproteins and LPL enzyme. It also plays a role in reverse cholesterol transport by inhibition of LCAT enzyme and the stimulation of CETP [32]. Mutations of the genes at this locus are commonly implicated in pathogenesis of several types of monogenic dyslipidemias. [31]. The association of the genetic variants of this *APOA-APOC3* cluster with serum lipid traits, including HDL-C, LDL-C and TC level was described in several GWAS across different populations worldwide [16, 20, 22–24, 26, 37, 38, 46, 47], and the variant rs964184 at this locus has been reported as the lead SNV in several studies [20, 23, 24, 26, 47].

#### APOA2

The *APOA2* gene at 1q23.3 locus encodes apolipoprotein A-II (apoA-II), which is a main structural component of HDL particles. Other than its role in cellular cholesterol efflux, apoA-II was proposed to impede reverse cholesterol transport by different mechanisms that include the inhibition of LCAT and CETP [48]. A role of apoA-II in regulation of LPL activity was demonstrated in studies on transgenic mice [49]. A cross-sectional survey in an Iranian population identified a missense mutation; Ala98Pro of the *APOA2* gene producing extreme HDL-C levels [50]. Genome-wide significant association of the polymorphism of *APOA2* gene with HDL particle size (HDL: medium particles) but not with HDL-C level was identified in the Caucasian population [22]. The association of *APOA2* polymorphisms (rs3813627, rs3829793) with plasma HDL-C levels was shown by candidate gene studies in which the majority of the participants were Caucasians [51, 52].

#### APOH

The *APOH* gene at the cytogenetic locus 17q24.2 encodes apolipoprotein H (apoH), which is also known as beta-2 glycoprotein 1. An in-vitro analysis has shown that apoH activates LPL enzyme in the presence of apoC-II [53]. It has also been reported that apoH blocks LDL oxidation and affects the intracellular cholesterol pool by reducing the influx and increasing the efflux of cholesterol in macrophages. Both these mechanisms contributes to the anti-atherogenic effect of apoH [54]. Significant association of a missense variant; Trp316Ser (rs1801690) of *APOH* gene with LDL-C level was observed among individuals with European ancestry in a large-scale meta-analysis of 32 studies [55]. Recently, this variant was also shown to be associated with TC and LDL-C levels in Chinese Malao and Han populations [56]. Association of another missense variant (rs1801689) in *APOH-PRXCA* locus with the LDL-C level was also described in a large-scale GWAS and meta-analysis [26].

#### APOM

Apolipoprotein M, encoded by *APOM* gene (locus 6p21.33) is primarily associated with HDL particles. It has been shown that apoM contributes to the atheroprotective role of HDL by modulating the size and function of the HDL particles [57]. Studies conducted in the Chinese population cohorts and meta-analyses have shown the association of APOM gene variants (rs707921, rs805296, rs805297) with serum lipid traits including HDL-C, LDL-C and TC levels [58–60], however none of the GWAS conducted to date has identified such association.

#### LPA

The *LPA* gene at 6q25.3-q26 encodes apolipoprotein(a) which is a major constituent of lipoprotein(a); a subclass of lipoprotein in plasma. Lipoprotein(a) consists of a LDL particle with the apolipoprotein(a) molecule bound via a disulfide molecule to apoB of the LDL. The link between the plasma lipoprotein-a level and the *LPA* variants with the cardiovascular diseases has already been established [61]. Recent GWAS and meta-analyses have shown the association of *LPA* gene variants; rs1564348 with plasma LDL-C and TC levels [23, 26] and rs1084651 with HDL-C level [23].

### Genes encoding lipoprotein receptors and related proteins

Lipoprotein receptors are involved in lipoprotein metabolism and the regulation of plasma lipid and cholesterol levels by receptor mediated uptake of lipoproteins. The strong association between LDL receptor (*LDLR*) gene mutations and autosomal dominant FH has already been established. In addition, the mutations in *LDLRAP1* gene encoding LDL receptor adaptor protein accounts for the autosomal recessive familial hypercholesterolemia. Few other lipoprotein receptor proteins with structural similarity to LDL receptor have been shown to be associated with serum lipid traits in GWAS and candidate gene studies.

#### LDLR

Majority of the patients with FH have a mutation in the *LDLR* gene at the 19p13.2 locus [6]. Five different types of mutations in the *LDLR* gene have been described according to the effect of the mutation on the function of the LDLR protein. These include 4 different types of mutations causing a defect in one of the 4 steps in LDL uptake and degradation pathway; transport, binding, internalization and recycling, and the fifth type which produces null-alleles with no receptor protein production [62]. To date nearly 1500 ‘pathogenic’ and ‘likely pathogenic’ variants of the *LDLR* gene are listed in the ClinVar database [63]. These includes a large number of single nucleotide substitutions producing nonsense, missense, frameshift or splice site variants or variants in the promoters or untranslated regions as well as copy number variants (CNVs) such as deletions, duplications, insertions and indels. These variants are distributed throughout the gene without clustering in any specific regions of the gene or the domains of the LDLR protein. [63, 64]. Several SNVs reaching the genome-wide significant level of association with the plasma LDL-C level were identified in GWAS that included diverse population groups [14–26, 38, 39] Among these SNVs, the association with rs6511720 polymorphism was replicated in several GWAS [15–17, 20, 23, 25, 26, 38] and in a candidate gene association study of a multiethnic population cohort in United States [65]. Other *LDLR* gene variants with replicated evidence of association in GWAS and candidate gene studies include rs688 [14, 27, 29], rs2228671 [18, 19], rs11668477 [18, 21] and rs1529729 [27, 65].

#### LDLRAP1

A rare autosomal recessive form of FH is produced by homozygous and compound heterozygous mutations of *LDLRAP1* gene at 1p36.11 locus. LDL receptor adaptor protein (LDLRAP) interacts with the LDL receptor during internalization process of LDL:LDL-receptor complex by endocytosis. Defective internalization process leads to defective LDL catabolism and elevation of plasma LDL-C levels producing the hypercholesterolemia phenotype [66]. Recent large-scale GWAS and meta-analyses have identified a *LDLRAP1* gene polymorphism (rs12027135) associated with plasma TC and LDL-C levels [23, 26]. A large-scale GWAS that included 16 European population cohorts showed the significant association between TC level and a variant (rs10903129) of the *TMEM57* gene which is at the same locus (1p36.11) [19].

#### LRP1

The *LRP1* gene at the locus 12q13.3 encodes LDL receptor related protein 1 (LRP1) which is involved in lipid metabolism by binding with apoE and the receptor mediated endocytosis of apoE containing lipoprotein particles [67]. The importance of circulating soluble LRP1 (sLRP1) protein level as a potential biomarker of hypercholesterolemia was indicated by the higher sLRP1 levels in patients with severe hypercholesterolemia compared to those with moderate elevation or normal levels of serum cholesterol [68]. *LRP1* gene variants are shown to be the predictors of cardiovascular disease risk in patients with FH [69]. The association of *LRP1* gene polymorphism (rs11613352) with HDL-C level was identified by recent GWAS and meta-analyses [23, 26].

#### LRP2

The LDL receptor related protein 2 (LRP2), also known as megalin is a member of the LDL receptor-related protein family. This protein is encoded by *LRP2* gene at 2q31.1 locus. It is involved in receptor mediated endocytosis of diverse range of ligands including lipoproteins. Its role in lipoprotein metabolism was further highlighted by the observation that LRP2 binds LPL [70, 71]. Genotyping of HDL-C candidate loci in a case–control cohort of individuals with extreme HDL-C levels and the meta-analysis with three replication cohorts have identified a missense variant; G669D (rs34291900) in *LRP2* gene which is significantly associated with HDL-C level [30].

#### LRP4

LRP4 is another member of the family of LDL receptor related proteins. The *LRP4* gene encoding this protein is mapped to 11p11.2 locus. Although the role of this protein in lipid or lipoprotein metabolism is not identified yet, a variant in this gene (rs3136441) was reported to be associated with plasma HDL-C level in GWAS and meta-analyses [23, 26].

#### LRPAP1

The *LRPAP1* gene (locus 4p16.3) encodes the LDL receptor-related protein associated protein 1 which is observed to have an inhibitory effect on ligand binding to LPR1 and LPR2 proteins [72]. A candidate gene study has reported that variants of the *LRPAP1* gene are associated with early onset myocardial infarction but not with the plasma lipid levels [73]. A recent GWAS on lipid traits and meta-analysis has shown an association of *LRPAP1* variant (rs6831256) with TC and LDL-C levels [26].

#### VLDLR

The VLDL receptor also belongs to the LDL receptor family of proteins. This receptor binds apoE containing lipoproteins, mainly VLDL and IDL. In-vitro studies have provided evidence of further involvement of this protein in lipid homeostasis by LPL catabolism in vascular endothelium [74] and up-regulation of ABCA1 increasing the cellular cholesterol efflux [75]. A large scale meta-analysis of studies done among Europeans has shown the association of *VLDLR* polymorphism; rs7024888 with LDL-C levels [55]. Furthermore, a recent GWAS and meta-analysis has shown the association of another variant; rs3780181 with TC and LDL-C levels [26].

#### SCARB1

The *SCARB1* gene mapped to chromosome 12 (12q24.31), encodes the scavenger receptor-B1 (SR-B1). This protein acts as a plasma membrane lipoprotein receptor and is involved in selective uptake of cholesteryl esters from HDLs and LDLs [76]. This protein is also supposed to be acting as a lipoprotein-a receptor [77] and also involved in LDL transcytosis in the vascular endothelium [78]. A study on transgenic mice identified the role of intestinal SR-B1 on cholesterol absorption [79], however these functions need further evaluation in humans. A SNV; rs838880 of *SCARB1* gene was shown to be associated with HDL-C level in GWAS [23, 26]. The most commonly studied *SCARB1* variant in candidate gene studies is rs5888, a synonymous variant which showed a significant association with HDL-C level [80–83] and some of these studies showed the gender and age specific association of this variant on plasma HDL-C level [80, 81, 83]. The association between another variant; rs11057851and HDL-C level was also observed in candidate gene association studies [84, 85].

### Genes encoding enzymes involved in lipid metabolism

#### Genes encoding enzymes involved in lipoprotein metabolism

##### *LPL*

The *LPL* gene at 8p21.3 locus encodes the LPL enzyme. It catalyzes the hydrolysis of triglycerides in chylomicrons and VLDL particles. Its role in receptor mediated cellular uptake of chylomicron remnants and lipoproteins are also described [86]. Several studies have reported that the mutations in *LPL* gene such as D9N and N291S cause an increase in total cholesterol and TG levels and a decrease in HDL-C level producing a hypercholesterolemia phenotype [87–90]. The association of SNVs in the *LPL* gene with the biochemical phenotype of hypercholesterolemia, primarily the HDL-C level was observed in many GWAS done in different population groups worldwide, with several SNVs having replicated evidence of association (e.g. rs12678919, rs328, rs10503669, rs17411031, rs10096633, rs17482753, rs2083637) [14, 16, 17, 19, 20, 22–26, 37, 38, 91–94].

##### ***LCAT***

The *LCAT* gene encodes lecithin: cholesterolacyl transferase (LCAT) enzyme. The action of this extracellular enzyme is to esterify the cholesterol taken up from the peripheral tissues. The esterified cholesterol molecules are then incorporated in to HDL particles for the transport to the liver. Mutations in *LCAT* gene can produce a hypercholesterolemia phenotype due to the impaired reverse cholesterol transport [95]. Several population-based studies have shown that the individuals with certain *LCAT* gene mutations or non-synonymous variants have a significantly low HDL-C level compared to those without the mutation or the variant [96–98]. In addition, common variants in the *LCAT* gene (e.g. rs255052, rs16942887) have been reported to be associated with HDL-C level in many populations [17, 20, 21, 23–26].

##### *SOAT1 and SOAT2*

Sterol O-acyltransferases (SOAT) are intracellular enzymes catalyzing the esterification of cholesterol, thereby involved in the chylomicrons assembly in enterocytes and the VLDL assembly in hepatocytes. Two isoforms of SOAT; namely SOAT1 and SOAT2 encoded by *SOAT1* gene (1q25.2) and *SOAT2* gene (12q13.13) respectively are described to-date [99]. The association of HDL-C level with a *SOAT1* gene haplotype with 3 variants (rs2783391, rs2247071 and rs2493121) was observed in Black Americans [100]. Another *SOAT1* variant; rs4421551 also showed association with HDL-C level in a population-based case control study in Caucasian population [30]. In Chinese Bai Ku Yao population, a *SOAT1* variant; rs1044925 has been shown to be associated with TC and LDL-C level, mainly in females [101]. A candidate gene analysis in a cohort of Caucasian individuals showed that *SOAT2* variant; rs2272296 is an independent predictor of HDL-C level [51]. However, none of the GWAS conducted to-date have identified SNVs of *SOAT1* or *SOAT2* genes that are associated with serum lipid traits.

##### *LIPC*

The *LIPC* gene at 15q21.3 locus encodes hepatic triglyceride lipase (HL); an enzyme catalyzing the hydrolysis of triglycerides and phospholipids in chylomicron remnants, IDL and HDLs. This process converts the IDL to LDL. It is also involved in receptor mediated uptake of lipoproteins by the hepatocytes [102]. The association between the serum lipid parameters and the common variants of the *LIPC* gene promoter region was extensively investigated during the last decade. The C250A, C514T and C480T variants of the *LIPC* promoter was shown to be associated with higher HDL-C levels in different populations [103–107]. Several SNVs associated with serum lipid parameters, mainly HDL-C level at a genome wide level of statistical significance were reported in multiple GWAS in different populations, and some SNVs (e.g. rs1532085, rs4775041, rs261332) showed evidence of association in more than one GWAS [14–17, 19–26, 37–39, 46, 94, 108].

##### *LIPG*

Endothelial lipase (EL) is coded by the *LIPG* gene at 18q21.1 locus. This protein is involved in the lipoprotein metabolism and the regulation of serum HDL-C level. It acts as a potent phospholipase than a triglyceride lipase and increases the cellular lipoprotein uptake [109, 110]. Studies on mouse models have demonstrated that EL affects the catabolism of apoB-containing lipoproteins [111]. Loss-of-function mutations in the *LIPG* gene have been shown to increase the HDL-C level indicating the role of EL in HDL metabolism [112]. Several candidate gene studies and meta-analysis have also shown that, a common non-synonymous SNV in *LIPG* gene, C584T is associated with elevated HDL-C levels in different populations [113–115]. Several SNVs of the *LIPG* gene associated with HDL-C and TC levels were reported in GWAS and meta-analyses of different populations, and rs2156552, rs4939883, rs7240405 and rs7241918 are among the lead SNVs with replicated evidence of association [15–17, 19, 20, 22–24, 26, 37, 39, 91, 92].

##### *LIPA*

The *LIPA* gene (locus 10q23.31) encodes the lysosomal acid lipase A that catalyzes the intracellular hydrolysis of cholesterol esters within LDL particles that are taken up by the hepatocytes [116, 117]. A splice site mutation of the *LIPA* gene (c.894G > A) was reported to be producing the hypercholesterolemia phenotype with elevated total cholesterol and LDL-C levels [118]. Variants in this gene (e.g. rs1412444 and rs2246833) were also found to be associated with hypercholesterolemia [119]. These findings indicates that *LIPA* is a candidate gene for the evaluation of inherited susceptibility to hypercholesterolemia, however no SNV with genome-wide significant association with serum lipid traits has been identified yet.

#### Genes encoding enzymes involved in cholesterol synthesis and catabolism

##### *HMGCR*

The *HMGCR* gene at 5q13.3 locus encodes the HMG-CoA reductase enzyme, which catalyze the conversion of 3-hydroxy-3-methylglutaryl CoA (synthesized from acetyl CoA) to mevalonate; the rate limiting step in cholesterol biosynthesis [120]. A promoter polymorphism (C911A; rs3761740) of the *HMGCR* gene was shown to be associated with TC level in Turkish males in a population-based case–control study [121], however the association was not observed in a candidate gene study of case–control design conducted in Western India [122]. Several GWAS and meta-analyses have identified multiple variants of *HMGCR* gene that are associated with TC and LDL-C levels [15, 16, 19, 20, 22–24, 26, 38, 94]. Among them rs3846662, rs12916 and rs12654264 showed replicated evidence of association in more than one GWAS and in candidate gene association studies [27, 60, 123].

##### *MVK*

Mevalonate kinase enzyme encoded by the *MVK* gene catalyzes the phosphorylation of mevalonate; which is the second most important step in cholesterol biosynthesis [120]. A study published in 2010 reported that the *MMAB* gene at the same locus as the *MVK* gene (12q24.11) might affect the HDL-C level [124], however, these is no role of the *MMAB* gene in lipid metabolism recognized to date. More recently, GWAS and meta-analyses have reported the association of plasma HDL-C level with other variants (rs7134594, rs4766613, rs9943753) at this locus [15, 23, 24, 26].

##### *CYP7A1*

The *CYP7A1* gene at 8q12.1 locus encodes the cholesterol 7α-hydroxylase enzyme which catalyzes the first step in cholesterol catabolism and the classical pathway of bile acid synthesis, the main mode of elimination of cholesterol from the body [125]. A family study has reported 3 individuals with a homozygous deletion mutation of *CYP7A1* gene leading to a frameshift (L413fsX414) and producing the hypercholesterolemic phenotype with elevated TC and HDL-C level [126]. A study in the Caribbean Hispanic population and two other studies conducted among Asian population cohorts have shown the association of *CYP7A1* variants (rs10957057, rs3808607) with TC and LDL-C levels [127–129]. Another *CYP7A1* variant (rs2081687) with genome-wide significant association with TC and LDL-C levels has also been identified in GWAs and meta-analyses [23, 26].

#### Genes encoding other enzymes involved in lipid metabolism and with evidence of association with serum lipid traits

Fatty acid desaturases (FADS) are a group of enzymes that are involved in the desaturation of fatty acids by forming carbon-carbon double bonds in the fatty acid chain [130]. Several different types of FADS enzymes are described in humans and FADS1, FADS2 and FADS3 enzymes are encoded by the ***FADS1,2,3*** gene cluster at 11q12.2 locus. GWAS have shown the association of several SNVs of this locus that are associated with lipid traits including TC, LDL-C and HDL-C levels [19–23, 25, 26]. Candidate gene association studies in European and Asian populations have confirmed this association with some of the common variants identified in GWAS (e.g. rs174537, rs174546, rs174547, rs174556) [131–133].

In a large-scale study that included seven population-based cohorts and meta-analysis, several non-synonymous and splice site variants in *PNPLA5* gene were shown to be associated with LDL-C level [134]. This gene at 22q13.31 locus encodes a member of a palatin-like phospholipase domain containing family of protein. Recently, in a study done on *PNPLA5*-knockout rats, it was shown that inhibition of *PNPLA5* expression leads to an elevation of TC, HDL-C and triglyceride level and a reduction of LDL-C level, giving more evidence for the involvement of this gene in lipid metabolism [135]. However no GWAS conducted as yet has identified variants of this gene that are associated with serum lipid traits.

A recent GWAS and meta-analysis has identified the variants associated with HDL-C levels in 2 other loci containing genes involved in glyceride synthesis and metabolism. These are the variants; rs702485 of *DAGLB* (diacylglycerol lipase, beta) gene at 7p22.1 and rs499974 of *MOGAT2* (monoacylglycerol O-acyltransferase 2) – *DGAT2* (diacylglycerol O-acyltransferase 2) locus at 11q13.5 [26].

### Genes encoding lipid transporters and lipid transfer proteins

#### Genes encoding ATP binding cassette (ABC) transporters family

The ATP binding cassette (ABC) proteins act as transporters of different substrates across membranes. Forty eight different types of ABC transporters have been identified in humans and many are implicated in diseases [136, 137]. Some of these transporters are involved in cholesterol transport and are reported to be associated with abnormal lipid profiles in humans.

The *ABCG5* and *ABCG8* genes at 2p21 locus are expressed in the enterocytes, hepatocytes and biliary endothelial cells. These transporters limit the intestinal absorption and promotes the biliary excretion of cholesterol, thereby reducing the plasma cholesterol level [138, 139]. The association of *ABCG5* or *ABCG8* gene variants with the clinical and biochemical phenotype of hypercholesterolemia was observed in several candidate gene studies in different populations [140–144]. Furthermore, few SNVs (rs4299376, rs6756629, rs6544713 and rs4245791) that are associated with plasma lipid traits, mainly LDL-C and TC level with a genome-wide significance was reported in several studies [19, 20, 22, 23, 26, 92].

ABCA1 transporter is involved in the cellular cholesterol efflux. The *ABCA1* gene encoding this transporter protein is mapped to chromosome 9 (9q31.1). Mutations in this gene are known to cause Tangier’s disease characterized by low serum HDL-C level [145]. The association of the *ABCA1* gene variants with the HDL-C level was shown in many GWAS and meta-analyses, and some variants (e.g. rs3890182, rs4149268, rs3905000, rs1883025) show replicated evidence of association [14–17, 19, 20, 22–26, 37–39, 108].

ABCG1 transporter is also involved in the cellular cholesterol efflux especially in macrophages. Up-regulation of *ABCG1* gene at 21q22.3 locus was observed to cause an increase in cholesterol efflux in to HDL particles [146]. A SNV (rs1893590) of *ABCG1* gene was reported to have a significant association with HDL-C level in an asymptomatic group of individuals in Brazil [147]. Another SNV (rs914189) at this locus with a significant association with HDL-C level in individuals of European ancestry was identified in a population-based case control study and meta-analysis [30].

The transport of bile salts synthesized from cholesterol in hepatocytes in to the biliary canaliculi occurs primarily via the ABCB11 transporter. Mutations in *ABCB11* gene (locus 2q31.1) encoding this protein causes progressive familial intrahepatic cholestasis [148]. A population based candidate gene association study in China showed significant association of *ABCB11* rs49550 variant with TC level [149]. A recent GWAS and meta-analysis has shown the association of another variant (rs2287623) of *ABCB11* gene with TC level [26].

In addition to ABCG5/8 and ABCB11, several other ABC transporter proteins are involved in the transport of substrates across the biliary canalicular membrane in to the bile for excretion. ABCB4 is involved in the transport of phosphatidylcholine in to the bile [150]. In a case–control study of patients with gallstones and healthy controls in Romania ABCB4 variants (e.g. rs1202283, rs31653) were shown to be associated with HDL-C level both in patients and controls [151]. ABCC2, another transporter in canalicular membrane, transports substrates such as drugs from hepatocytes to the bile [152]. Although its role in lipid transport is not fully recognized, one study has reported an indel/splice site variant of the *ABCC2* gene (g.101591890dup) that is associated with a low HDL-C level [153]. Association between the variants of these genes and serum lipid traits were not observed in any of the GWAS conducted to date.

A candidate gene association study in which the majority of subjects were Caucasians, has shown the association of SNVs of another ABC transporter gene; *ABCC6* (rs150468 and rs212077) with low HDL-C level [52]. ABCC6 transporter was localized to the basolateral membrane of hepatocytes indicating its role in transport between sinusoids and hepatocytes [154]. Recently an in-vitro study showed that ABCC6 deficiency leads to increased cholesterol synthesis and reduced expression of *PCSK9* and *APOE* genes [155].

ABCA8 is expressed in many organs in the human body including the liver, however its function is not clearly recognized yet [156]. A candidate gene association study of case–control design in Dutch population has identified an indel variant (c.2219_2220dup) of *ABCA8* gene that is associated with low HDL-C level [153]. Recent GWAS and meta-analyses have reported the association of *ABCG8* variant; rs4148008 with HDL-C level [23, 26].

##### *NPC1L1*

The *NPC1L1* gene at 7p13 locus encodes Niemann-Pick C1-like 1 protein which is a transmembrane protein involved in the absorption of dietary cholesterol in the intestine and the absorption of cholesterol in the biliary canalicular lumen back in to the hepatocytes [157]. It has been shown that the low intestinal cholesterol absorbers are more likely to have non-synonymous variants of the *NPC1L1* gene than the high absorbers [158]. The association of *NPC1L1* gene variants with the TC and LDL-C level was observed in candidate gene association studies [159–161]. These variants include rs2072183, rs17655652, rs41279633, rs217434 and rs3187907. Among them, rs2072183 showed the association with TC and LDL-C level also in GWAS with meta-analyses [23, 26].

##### *CETP*

The *CETP* gene (16q13) encodes the plasma protein; CETP which is involved in the transfer of cholesteryl esters from HDL particles to other lipoproteins such as VLDL, IDL and LDL. This process is important in reverse cholesterol transport from peripheral tissues to the liver [162]. Significant association between elevated CETP level and high total cholesterol and LDL-C levels and low HDL-C levels was observed among individuals with hypercholesterolemia [163, 164]. The association of common variants of *CETP* gene with the serum lipid traits, especially HDL-C level was observed in many GWAS and meta-analysis across different populations. Among them rs3764261 was identified as the lead SNV in majority of these studies and few other SNVs (rs9989419, rs247617, rs1800775) showed the association in more than one study [14–17, 19–26, 37–39, 46, 47, 91–94, 108, 165, 166].

##### *PLTP*

Phospholipid transfer protein (PLTP) encoded by the *PLTP* gene at 20q13.12 locus is involved in phospholipid transfer from triglyceride rich lipoproteins to the HDLs. This process contributes to the formation of LDLs from the VLDL particles as well as the maturation of HDLs [167]. Candidate gene studies have shown the association of *PLTP* gene variants (e.g. rs378114, rs2294213) with HDL-C levels [30, 168, 169]. Genome-wide significant association of *PLTP* variants (rs7679, rs6065906, rs6065904) with plasma lipid traits, mainly HDL-C level were also observed in several studies [20, 22, 23, 25, 26, 37].

#### Genes encoding OSBP-like (OSBPL) proteins

Oxysterol-binding protein (OSBP)-related protein family consists of several intracellular lipid-binding proteins in humans. These proteins are involved in lipid transfer by facilitating the movement of lipids between membranes, transient addition or removal of lipids from membranes, regulating the binding of membrane lipids with other lipid-binding proteins or by their action as lipid sensors. Recent evidence from animal studies have shown the role of ORPs in cholesterol transfer, cellular cholesterol efflux and regulation of ABCA1 expression [170]. Recent GWAS and meta-analyses have shown the association of *OSBPL7* variant; rs7206971 with the LDL-C and TC level [23, 26] and the association of another variant; rs17259942 at *OSBPL8-ZDHHC17* locus with HDL-C level [92]. A candidate gene study with a case control design in Dutch population showed the association of an indel variant (c.109_112dup) of *OSBPL1A* gene with low HDL-C level and a nonsense variant (c.145C > T) of *OSBPL3* gene with high HDL-C level [153].

#### STARD3

The *STARD3* gene at 17q12 locus encodes a steroidogenic acute regulatory protein (StAR)-related lipid transfer domain-containing protein, which is involved in intracellular cholesterol trafficking from lysosomes to the mitochondria [171]. Recent GWAS and meta-analyses have shown the association of *STARD3* polymorphism (rs11869286) with serum HDL-C level [23, 26].

### Genes encoding proprotein convertases

Proprotein convertase family consists of several types of proteins that are involved in many important biological processes in humans including lipoprotein metabolism. Though their function varies according to their location, in general these proteins bring about their effect by the activation of other proteins [172].

The *PCSK9* gene at 1p32.3 locus is expressed primarily in the liver and encodes proprotein convertase subtilisin/kexin type 9 (PCSK9). This protein plays an important role in LDL catabolism by escorting the LDL:LDL-receptor complex for lysosomal degradation [173]. Gain-of-function mutations of *PCSK9* gene causes an increase in LDL receptor degradation resulting in a reduction in the LDL receptor expression on the cell surface. This leads to accumulation of LDL levels in plasma giving rise to hypercholesterolemia and is described as a rare, yet well-known cause of autosomal dominant FH [6]. Several GWAS and meta-analyses have also shown the association of *PCSK9* variants, commonly rs11206510 and rs2479409, with the plasma lipid traits, primarily the LDL-C levels [15, 17, 20, 22–26, 39]. Association *PCSK9* variants with LDL-C level was also reported in candidate gene studies [27, 29].

The *PCSK5* at 9q21.13 is another member of this family which is known to play a role in lipoprotein metabolism, probably by inactivation of LPL and EL enzymes [174]. Few candidate gene association studies have shown the association between *PCSK5* polymorphisms (e.g. rs1340510, rs11144782, rs11144766) and plasma lipid traits, primarily HDL-C levels [30, 153, 175]. Significant association of *PCSK6* (15q26.3) variant (rs1471656) with HDL-C level was also identified in a study done in a Caucasian population [30], however the effect of this protein on lipid metabolism has not yet been clearly identified. *PCSK8* or *MBTPS1* gene at 16q23.3-q24.1 locus encodes the membrane bound transcription factor peptidase, site 1 which is known to be involved in the regulation of cholesterol metabolism [176, 177]. However, none of the GWAS conducted to date showed the association of *PCSK5*, *PCSK6* or *PCSK8* polymorphism with serum lipid traits.

### Other genes encoding proteins with an established or potential role in lipid metabolism

Many other genes that encode proteins involved in the regulation of lipid/cholesterol or lipoprotein function and metabolism have been identified. Variants in some of these genes showed significant association with serum lipid traits including TC, HDL-C and LDL-C levels. The association of some of these variants was replicated in several GWAS and candidate gene association studies.

#### SORT1

The *SORT1* gene encodes sortilin which is involved in the cholesterol homeostasis in humans. Sortilin increases the hepatic output of VLDL which acts as a precursor for LDL in plasma. This protein also enhances the secretion of PCSK9 from the hepatocytes, which will cause LDL receptor degradation. Both these actions of sortilin leads to an elevation of plasma LDL-C level [178, 179]. More recently, it was also shown that sortilin reduces the apolipoprotein-B secretion by the liver [180]. Several candidate gene studies have identified significant association of *SORT1* gene variants with LDL-C level in different populations [65, 181–185], and some SNVs were reported to have an age and sex specific effect on LDL-C level [186, 187]. Two other genes; *CELSR2* and *PSRC1* are mapped to the same locus as the *SORT1* gene at 1p13.3. The association of SNVs at this locus with plasma lipid traits especially with LDL-C and TC levels was further reinforced by several GWAS [14–26, 38, 92, 166]. Among these, rs646776, rs599839, rs12740374 and rs629301 variants showed replicated evidence of association. A functional study on *SORT1* variants has suggested that rs12740374 affects the hepatic expression of the gene, by either creating (minor allele) or disrupting (major allele) a binding site for CCAAT/enhancer-binding transcription factors [188].

#### Genes encoding angiopoietin-like proteins (ANGPTL)

Angiopoietin-like proteins (ANGPTL) family has been shown to have diverse biological functions other than their role in angiogenesis. Some of these proteins (ANGPTL1, ANGPTL3, ANGPTL4, ANGPTL6 and ANGPTL8) are primarily expressed in the liver and some (ANGPTL3, ANGPTL4 and ANGPTL8) are well-recognized to be involved in lipoprotein metabolism [189]. These 3 proteins regulates the triglyceride metabolism by tissue-specific inhibition of LPL activity in fed and fasting states. In the fed state, increased ANGPTL8 expression leads to activation ANGPTL3. ANGPTL3 inhibits LPL in cardiac and skeletal muscles directing triglycerides to adipose tissue for storage. Fasting induces ANGPTL4; an inhibitor of LPL in adipose tissue delivering triglycerides to other tissues including cardiac and skeletal muscles for oxidation [190]. It has also been shown that ANGPTL3 inhibits the activity of EL [191] and HL [192]. A recent study has also shown that reduced *ANGPTL3* expression leads to a reduction in LDL production by increasing the clearance of apoB containing lipoproteins (but not LDL or β-VLDL) by a pathway not yet recognized. This reduces the VLDL fraction that is available for the production of LDL [193]. *ANGPTL3* gene is mapped to chromosome 1p31.3 and *ANGPTL8* and *ANGPTL4* genes to the chromosome 19p13.2. An *ANGPTL3* polymorphism; rs11207997 has been shown to be associated with a lower HDL-C level in a study that included individuals from several European countries [194]. The association of a variant in *ANGPTL8* gene (rs2278426) with lower TC and HDL-C level was observed in American Indians and Mexican Americans [195] and the males of the Chinese Han population [196]. Conversely, higher HDL-C level was observed in the carriers of 40 K variant (‘A’ allele) of the commonly studied E40K variant (118G > A) of ANGPTL4 gene [197–199]. Recent GWAS and meta-analyses have shown the association between *ANGPTL3* rs2131925 variant with LDL-C and TC, and *ANGPTL8* rs737337 and *ANGPTL4* rs7255436 variants with HDL-C levels [23, 26]. Few other variants with genome-wide significant association are described in the *ANGPTL3-DOCK7* locus (rs10889353, rs1167998, rs11207995) for LDL-C and TC levels [15, 19, 25, 94], and in the *ANGPLT4* locus (rs2967605) for HDL-C level [20]. The association of a variant (rs4650994) of *ANGPTL1* gene at 1q25.2 locus with HDL-C level was also observed in a GWAS and meta-analysis [26], however the role of this gene in lipid metabolism has not been identified yet. *ANGPTL6* gene is another member of the ANGPTL gene cluster at 19p13.2 locus. In a cohort of Korean individuals with metabolic syndrome, serum ANGPTL6 level was significantly higher in patients with low HDL-C level [200]. The association of the ANGPTL6 gene with serum lipid traits was also observed in a study done on transgenic mice, in which the Angptl6 deficient (Angptl−/−) mice showed significantly elevated serum cholesterol level [201]. Further investigations are required to identify the association of *ANGPTL6* variants with serum lipid traits and to define the role of this gene in lipid metabolism in humans.

#### GALNT2

O-linked glycosylation is a type of post-translational modification that affects the expression, structure, stability, processing or the functions of the protein. The N-actetylgalactosaminyltransferase 2 protein encoded by *GALNT2* gene at 1q42.13 locus catalyzes the first step of O-glycosylation. Several proteins involved in lipid metabolism such as ApoA-II, LCAT, LDL receptor, VLDL receptor have an established O-glycosylation site. It has also been predicted that several other proteins; CETP, PLTP, SCARB1, EL and ANGPTL3 also have an O-glycosylaion site, highlighting the importance of GALNT2 in lipid metabolism [202]. The association of *GALNT2* polymorphism with HDL-C level was observed in several GWAS and meta-analyses, and the variants; rs4846914 and rs2144300 showed replicated evidence of association [16, 17, 20, 23–26, 38]. The association between rs4846914 variant and HDL-C was further established by the findings of candidate gene association studies conducted in an Asian Malay population in Singapore [203], in Mexican population [204], and in a multiethnic cohort in United States [65]. A population-based case–control study and meta-analysis confirmed the association of rs2144300 with HDL-C level [30]. Studies conducted in different Chinese populations also confirmed the association of rs4846914, and identified some other *GALNT2* variants (rs4846913, rs2760537, rs1997947, rs11122316) with hypercholesterolemia and serum lipid traits [205, 206].

Variants of two other genes encoding galactosyltransferases (beta-1,3-galactosyltransferase 4 by *B3GALT4* gene at 6p21.32, and beta-1,4-galactosyltransferase 4 by *B4GALT4* gene at 3q13.32) were observed to be associated with LDL-C level in GWAS. These variants are *B3GALT4* rs2269346 [15], and *B3GALT4* rs2254287 and *B4GALT4*, rs12695382 [17].

#### MYLIP

The *MYLIP* gene mapped to chromosome 6p22.3 codes for inducible degrader of LDL receptor (IDOL). This protein, along with PCSK9 reduces the expression of LDL receptors on cell surface by stimulating LDL-receptor internalization and lysosomal degradation. This leads to accumulation of LDL in plasma increasing the LDL-C level [207]. The association of a SNV at *MYLIP-GMPR* locus (rs2142672) with LDL-C level was identified in a GWAS in individuals of European descent [24]. Two large-scale GWAS and meta-analyses reported the association of another variant; rs3757354 with the LDL-C level [23, 26]. A candidate gene association study in two ethnic groups in China showed the association of this variant with serum TC, and HDL-C levels [208]. Another non-synonymous variant; rs9370867 of *MYLIP* gene was shown to be associated with TC and LDL-C levels in candidate gene studies conducted in Mexican [209] and Italian [210] populations.

#### GCKR

The *GCKR* gene mapped to chromosome 2p23.3 encodes glucokinase regulatory protein which is primarily expressed in the liver. This protein acts as an inhibitor of glucokinase enzyme, thereby plays a key role in glucose and lipid metabolism [211]. Several GWAS showed the association of *GCKR* variants primarily with serum TG level [15, 16, 19, 20, 22, 23, 26], moreover two of these GWAS and meta-analyses showed the association of the GCKR rs1260326 variant with plasma TC level [23, 26]. Homozygosity for the ‘T’ allele of rs1260320 was also shown to be associated with elevated TC and LDL-C levels in a Polish group of children with monogenic diabetes mellitus and type-1 diabetes mellitus [212]. In a Korean population-based candidate gene study, two other *GCKR* variants (rs780094 and rs780092) showed the association with TC level in adults and TC and LDL-C level in children [213].

#### PON1,2,3

Paraoxonase gene cluster encoding *PON1, PON2 and PON3* genes is located at 7q21.3. Paraoxonases have several lipid and lipoprotein related functions contributing to their atheroprotective effect. These include reduced cholesterol synthesis and reduced LDL uptake by macrophages, increased cholesterol efflux and prevention of LDL and HDL oxidation [214]. Few variants of the *PON1* gene was commonly investigated for their association with serum lipid traits. These variants include rs662 (Q192R), rs854560 (L55 M) and rs705379 which showed the association with TC, HDL-C and LDL-C in different populations worldwide [215–219]. However none of the GWAS conducted to date has identified the association of the polymorphism at this locus with serum lipid traits.

#### NCAN, CILP2, PBX4

Many GWAS and candidate gene association studies in different populations have identified the association of the genetic variants of *NCAN, CILP2, PBX4* locus at 19p13.11 with serum lipid traits. The rs10401969 was identified in GWAS and meta-analyses as the lead SNV of this locus that is associated with serum lipid traits, primarily TC and LDL-C level [20, 23, 24, 26]. The genome-wide significant association of *NCAN, CILP2, PBX4* rs16996148 variant with LDL-C level was identified in two other GWAS [16, 17] while another variant; rs2304130 was show to be significantly associated with TC level in multiple European population cohorts in a GWAS [19]. The association of rs16996148 variant with serum lipid traits observed commonly in the Western populations in GWAS was replicated in Asian populations in candidate gene association studies, which showed the association of this variant with TC, LDL-C and also with HDL-C levels [203, 220]. Despite the evidence of association of these genetic variants with serum lipid concentration in many studies, the role of the genes at this locus in lipid metabolism is not yet defined. It has been demonstrated that proteoglycans, by binding to apoE secreted by macrophages enhances the macrophage cholesterol efflux [221]. Neurocan, encoded by *NCAN* is also an abundant proteoglycan in extracellular matrix, but its effects on lipid metabolism warrants furthers investigation.

Genome-wide association studies provided replicated evidence of association between the serum lipid traits and the variants of two other genes; *PPP1R3B* (8p23.1) and *TTC39B* (9p22.3) with a potential role in lipid metabolism. Genome-wide significant association of the *PPP1R3B* variants; rs9987289 with HDL-C, LDL-C and TC levels [23, 25, 26], and rs2126259 with LDL-C level [24] have been observed. A candidate gene study in Chinese Han population also showed the association of these 2 variants with LDL-C and TC levels and another *PPP1R3B* variant; rs19334 with LDL-C level [222]. Few SNVs in *TTC39B* gene associated primarily with HDL-C level have been identified in GWAS and meta-analyses. These variants include rs471364 [20], rs581080 [23, 26] and rs643531 [24]. Functional studies in mouse models have demonstrated the role of these two genes in lipid metabolism [23], however further investigations are required to identify the precise role of these genes with regard to lipid homeostasis.

Figure 1 presents an outline of the pathways of cholesterol and lipoprotein transfer and metabolism and the genes involved in these processes.

### Genes encoding transcription regulators of the genes involved in lipid metabolism

#### *SREBF1* and *SREBF2*

Sterol regulatory element binding factors (SREBFs) act as transcriptional regulators of many proteins involved in lipid metabolism. These includes *LDLR, HMGCR, LPL* [223], *LRP1* [224], *ABCA1* [225], *NPC1L1* [226], *PCSK9* [227] and *LIPG* [228]. Association between the *SREBF1* gene variants and the plasma cholesterol level was observed in candidate gene association studies [229, 230]. Similar association was observed with the *SREBF2* gene polymorphisms with LDL-C and TC levels [231–233]. However, none of the GWAS conducted to date, identified SNVs of these two genes with a significant association with lipid traits.

#### SCAP

The SREBP cleavage activating protein (SCAP) encoded by the *SCAP* gene escorts the SREBFs from the endoplasmic reticulum to the Golgi apparatus, where the SREBFs get cleaved and activated. The binding of SCAP to SREBF is dependent on the availability of cholesterol [234]. Homozygosity for a missense variant in *SCAP* gene was shown to be associated with higher LDL-C level in a group of individuals with hypercholesterolemia from Israel, Netherlands and Switzerland [233].

#### Insulin induced gene 2 (*INSIG2*)

The INSIG proteins regulate the cholesterol synthesis via SCAP-SREBF pathway. In the presence of cholesterol INSIG binds with SCAP and prevents the transport of SREBF to Golgi for its activation. In the absence of activated SREBF, transcription of genes involved in cholesterol biosynthesis is halted exerting a negative feedback effect [234]. Few candidate gene studies in different populations have identified the association of SNVs of *INSIG2* gene (e.g. rs12464355, rs7566605) with hypercholesterolemia and serum lipid traits, especially LDL-C level [235–238]. A recent GWAS and meta-analysis showed the association of *INSIG2* gene variant (rs10490626) with TC and LDL-C levels [26].

#### Hepatocyte nuclear factor (*HNF*) genes

Hepatocyte nuclear factors is a family of transcription factors regulating the transcription of many genes including those involved in lipid metabolism [239]. For example *NPC1L1* gene promoter region has a binding site for HNF1 alpha protein [240] which is encoded by *HNF1A* gene at 12q24.31 locus. The potential role of HNF1 alpha protein on *PSCK9* gene transcription has also been described [241]. The association of SNVs of the *HNF1A* gene (e.g. rs1169288, rs2650000) with LDL-C and TC level was shown in GWAS [20, 22, 23, 26]. The *HNF4A* gene is mapped to the chromosome 20 (20q13.12). HNF4 alpha protein acts as a regulator of *HNF1A* gene transcription [239]. The association of a *HNF4A* gene variant (rs1800961) with HDL-C and TC level was identified in GWAS and meta-analyses [20, 22, 23, 26]. This association was further established by the observation of a candidate gene study which showed significant association with HDL-C in a Pima Indian population cohort [242].

#### *NR1H3* – Liver X receptor alpha

The *NR1H3* gene at 11p11.2 codes for one of the two isoforms of liver X receptors (LXR). The other isoform, LXR-beta is encoded by *NR1H2* gene at 19q13.33. LXRs are involved in cholesterol homeostasis in by acting as receptors for the products of cholesterol oxidation within the cell and regulating the transcription of genes involved in cholesterol metabolism. LXRs up-regulate a number of genes involved in cholesterol excretion in liver and intestine, cholesterol efflux from peripheral tissues and reverse cholesterol transport such as *ABCG5, ABCG8, ABCA1, ABCG1, CYP7A1* and *PLTP*. It has also been shown that LXRs down-regulate *NPC1L1* gene reducing the intestinal cholesterol absorption. All these actions contributes to the reduction of intracellular cholesterol load [243]. Two other genes are mapped to the 11p11.2 locus; *MADD* and *FOLH1*. The association of variants at this locus (rs7120118, rs2167079 and rs7395662) with serum HDL-C level was observed in GWAS that included European and Asian population groups [19, 21, 38].

#### TRIB1

The *TRIB1* gene at 8q24.13 encodes the tribbles pseudokinase 1. A study conducted on transgenic mouse models has shown an inhibitory effect of TRIB1 on expression of lipogenic enzymes [244] and it was suggested that TRIB1 down-regulates the transcription of these lipogenic genes in humans [245]. GWAS and meta-analyses have shown the association between TRIB1 variants with serum lipid traits including TC, LDL-C and HDL-C levels, among these rs2954029 variant showed replicated evidence of association [15, 19, 23, 24, 26]. A candidate gene association study replicated the association of this variant with serum LDL-C level in the Danish population [246]. Another TRIB1 variant; rs17321515 was shown to be associated with TC, LDL-C and HDL-C in different populations in Asia [186, 204, 247].

#### Genes encoding peroxisome proliferator activated receptors (PPAR)

A recent GWAS and meta-analysis has identified a variant (rs4253772) of the *PPARA* gene at 22q13.31 locus associated with TC and LDL-C levels [26]. This gene encodes PPAR-alpha protein, which is a member of the family of nuclear receptors and one of the three subtypes of PPAR; PPAR-alpha, PPAR-gamma and PPAR-delta. Fatty acids and fatty acid derivatives acts as ligands to these receptor proteins. PPAR-alpha and PPAR-gamma form heterodimers with retinoid X receptors (RXR) and upon ligand binding these heterodimers interact with the promoters of the target genes regulating their transcription. PPAR-delta acts as an inhibitor of the activity of PPAR-alpha and PPAR-gamma proteins [248–250]. PPAR-alpha has an established or a potential role in up-regulation of *ABCA1, LPL, LIPC, LCAT, LTLP, ABCA1* and *ANGPTL4* genes, and down-regulation of *APOC3, CETP* and *SCARB1* genes; all with recognized roles in lipoprotein metabolism [248]. The *PPARG* gene at the 3p25.2 locus encodes PPAR-gamma protein which is primarily expressed in adipose tissue. This protein is involved in the transcriptional regulation of genes involved in lipid uptake and storage in adipocytes [249]. PPAR-delta protein encoded by *PPARD* gene at 6p21.31 locus regulates the expression of genes involved in fatty acid oxidation [250]. Candidate gene studies have identified the association of the variants in *PPARA* (e.g. rs1800206), *PPARG* (e.g. rs1805192, rs10865710) and *PPARD* (e.g. rs9794) genes with the serum lipid traits [248–253].

#### Genes encoding retinoid X-receptors (RXR)

Retinoid X-receptors (RXR) are a family of nuclear receptors with three subtypes, RXR-alpha, RXR-beta and RXR-gamma. These proteins acts as transcription factors by forming heterodimers with other nuclear receptors such as PPARs [254]. RXR-beta is encoded by *RXRB* gene at 6p21.32 locus which also includes *B3GALT4* gene. A SNV at this locus; rs2269346 was shown to be significantly associated with LDL-C level in a GWAS [15].

#### MLXIPL

The *MLXIPL* gene encodes MLX interacting protein like protein which acts as a transcription factor for the genes involved in glucose and lipid metabolism. This protein partnering with other transcription factors such as carbohydrate response element binding protein, has been shown to stimulate the transcription of lipogenic genes by binding the carbohydrate response element of the promoter region of these genes [255]. Recent GWAS and meta-analyses have shown the association of a *MLXIPL* variant; rs17145738 with serum HDL-C level [23, 26].

**Fig. 2:**
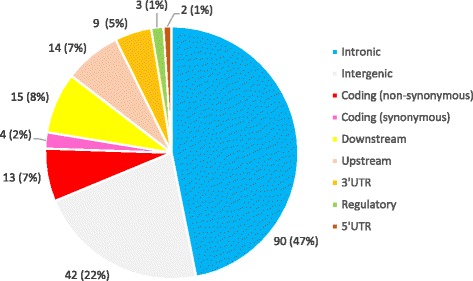
Types of TC, LDL-C, HDL-C-associated SNVs in genes with a recognized/potential role in lipid metabolism

In summary, GWAS and meta-analyses have identified over 190 SNVs associated with serum TC, LDL-C and HDL-C levels in about 60 genes (Additional file 1: Table S1). Annotation of these variants against Ensembl [256] and RefSeq [257] databases using SNPnexus [258] showed that only 9% of these variants are located within the coding regions of the genes. Fourteen non-synonymous coding variants were identified in 10 genes that accounts for 7% of the variants in lipid related genes described in GWAS. These genes are *APOB, APOE, APOH, LPL, CETP, ABCG5/8, PCSK9, GCKR, HNF1A* and *HNF4A* Majority of the variants are in the intronic regions of the genes (47%) and in intergenic regions (22%) (Fig. 2). Candidate gene association studies have identified SNVs in several other lipid-related genes that have not been identified in GWAS conducted to-date.

## Other loci associated with plasma lipid levels identified by GWAS

**Fig. 3:**
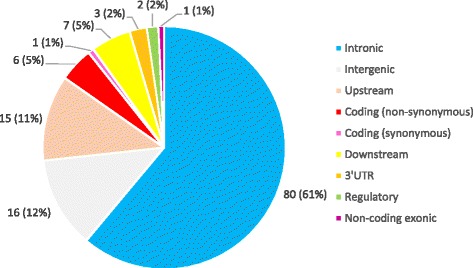
Types of TC, LDL-C, HDL-C-associated SNVs in genes with no recognized role in lipid metabolism

Recent advances in sequencing technologies (next-generation sequencing) and the bioinformatics tools allow the rapid sequencing and analysis of the whole genome or the exons of the individuals and permits large scale population-based surveys of genetic variation. This has contributed greatly to the improvement of our understanding of the genetic aetiology of the complex disorders such as polygenic hypercholesterolemia. During the last 10 years there were many GWAS that have identified many SNVs associated with the different serum lipid traits and hypercholesterolemia. Additional file 2: Table S2 presents a list of SNVs with genome-wide significance that influence the serum lipid levels in different populations which are not discussed earlier in this review. Most of the loci listed in this table are within or in the vicinity of the genes that are not known to be involved in lipid metabolism. Among these SNVs, majority (61%) are located in the intronic regions. Coding SNVs accounts for only 6% of these variants identified GWAS, and there are only 6 non-synonymous variants (Fig. 3). This indicates that there might be a considerable number of unrecognized processes and mechanisms causing dysregulation of lipid homeostasis and hypercholesterolemia. These variants need to be assessed further for the replication of the identified association in different populations. Moreover, functional studies has to be carried out to recognize the pathogenic mechanism of these variants with regard to hypercholesterolemia and serum lipid traits.

## Conclusion

Although there are major advances in the evaluation of genetic susceptibility of hypercholesterolemia during the last decade, significant challenges remain. The known genes account for only a proportion of the cases and a substantial amount of missing heritability still exists. Most of the identified genetic variants have a small effect and in combination do not explain much of the heritability of this complex disorder. Additionally, majority of the genetic variants that were shown to influence the plasma lipid levels and determine the inherited susceptibility to hypercholesterolemia are located outside the coding regions of these genes, hence will be missed in routine exome-sequencing techniques. Considering the association of hypercholesterolemia with more debilitating cardiovascular diseases such as coronary artery diseases and stroke, there is a need of further evaluation of the molecular genetic basis of this condition. Identification of new molecular mechanisms and pathways by this approach will provide new insights into the identification of novel treatment and preventive methods, as well as the identification and development of new biochemical and molecular markers for screening and monitoring. This will allow the early detection of asymptomatic patients and effective treatment, thus preventing complications and reducing the mortality and morbidity associated with this common disorder.

## Additional files

Additional file 1: Table S1. SNPs in the genes with a recognized or potential role in lipid metabolism, identified to be associated with TC, LDL-C and HDL-C levels in GWAS. (XLSX 29 kb)

Additional file 2: Table S2. SNVs in the genes with no recognized role in lipid metabolism, identified to be associated with TC, LDL-C and HDL-C levels in GWAS. (XLSX 16 kb)

## Abbreviations

ABC: ATP binding cassette protein
ANGPTL: Angiopoietin-like protein
Apo: Apolipoprotein
CETP: Cholesteryl ester transfer protein
CNV: Copy number variant
EL: Endothelial lipase
FAD: Fatty acid desaturase
FH: Familial hypercholesterolemia
GWAS: genome-wide association studies
HDL: High density lipoprotein
HDL-C: HDL cholesterol
HL: Hepatic lipase
HNF: Hepatocyte nuclear factor
IDL: Intermediate density lipoprotein
IDOL: Inducible degrader of LDL receptor
INSIG: Insulin-induced gene
LCAT: Lecithin: cholesterolacyl transferase
LDL: Low density lipoprotein
LDL-C: LDL cholesterol
LDLRAP: LDL receptor adaptor protein
LPL: Lipoprotein lipase
LRP: LDL receptor related protein
LXR: Liver X receptor
ORP: Oxysterol binding protein-related protein
OSBPL: Oxysterol binding protein-like protein
PCSK: Proprotein convertase subtilisin/kexin protein
PLTP: Phospholipid transfer protein
PPAR: Peroxisome proliferator activated receptor
RXR: Retinoid X receptor
SCAP: SREBF cleavage activating protein
sLRP1: Soluble LDL receptor related protein 1
SNV: Single nucleotide variant
SOAT: Sterol O-acyltransferase
SR-B1: Scavenger receptor B1
SREBF: Sterol regulatory element binding factor
TC: Total cholesterol
VLDL: Very low density lipoprotein

## References

[1] Global Health Observatory. Geneva: World Health Organization. http://www.who.int/gho/en/. Accessed 10 Jan 2017.

[2] Prospective Studies Collaboration (2007). Blood cholesterol and vascular mortality by age, sex, and blood pressure: a meta-analysis of individual data from 61 prospective studies with 55 000 vascular deaths. Lancet.

[3] Law MR, Wald NJ, Rudnicka AR (2003). Quantifying effect of statins on low density lipoprotein cholesterol, ischaemic heart disease, and stroke: systematic review and meta-analysis. BMJ.

[4] Global Health Estimates 2013 (2014). Deaths by cause, age and sex; estimates for 2000–2012.

[5] Projections of mortality and causes of death, 2015 and 2030. Geneva: World Health Organization. http://www.who.int/healthinfo/global_burden_disease/projections/en/. Accessed 10 Jan 2017.

[6] Soutar AK, Naoumova RP (2007). Mechanisms of disease: genetic causes of familial hypercholesterolemia. Nat Clin Pract Cardiovasc Med.

[7] Youngblom E, Pariani M, Knowles JW. Familial Hypercholesterolemia. 2014 Jan 2 [Updated 2016 Dec 8]. In: Pagon RA, Adam MP, Ardinger HH, et al., editors. GeneReviews® [Internet]. Seattle (WA): University of Washington, Seattle; 1993–2017. https://www.ncbi.nlm.nih.gov/books/NBK174884/. Accessed 10 Jan 2017.

[8] Marques-Pinheiro A, Marduel M, Rabès JP, Devillers M, Villéger L, Allard D (2010). A fourth locus for autosomal dominant hypercholesterolemia maps at 16q22. 1. Eur J Hum Genet.

[9] Fouchier SW, Dallinga-Thie GM, Meijers JC, Zelcer N, Kastelein JJ, Defesche JC (2014). Mutations in STAP1 are associated with autosomal dominant hypercholesterolemia. Circ Res.

[10] Varret M, Abifadel M, Rabès JP, Boileau C (2008). Genetic heterogeneity of autosomal dominant hypercholesterolemia. Clin Genet.

[11] De Castro-orós I, Pocoví M, Civeira F (2013). The fine line between familial and polygenic hypercholesterolemia. Clin Lipidol.

[12] Vrablik M, Ceska R, Horinek A (2001). Major apolipoprotein B-100 mutations in lipoprotein metabolism and atherosclerosis. Physiol Res.

[13] Andersen LH, Miserez AR, Ahmad Z, Andersen RL (2016). Familial defective apolipoprotein B-100: a review. J Clin Lipidol..

[14] Wallace C, Newhouse SJ, Braund P, Zhang F, Tobin M, Falchi M (2008). Genome-wide association study identifies genes for biomarkers of cardiovascular disease: serum urate and dyslipidemia. Am J Hum Genet.

[15] Chasman DI, Paré G, Zee RY, Parker AN, Cook NR, Buring JE (2008). Genetic loci associated with plasma concentration of low-density lipoprotein cholesterol, high-density lipoprotein cholesterol, triglycerides, Apolipoprotein A1, and Apolipoprotein B among 6382 white women in genome-wide analysis with replication, clinical perspective. Circ Cardiovasc Genet.

[16] Kathiresan S, Melander O, Guiducci C, Surti A, Burtt NP, Rieder MJ (2008). Six new loci associated with blood low-density lipoprotein cholesterol, high-density lipoprotein cholesterol or triglycerides in humans. Nat Genet.

[17] Willer CJ, Sanna S, Jackson AU, Scuteri A, Bonnycastle LL, Clarke R (2008). Newly identified loci that influence lipid concentrations and risk of coronary artery disease. Nat Genet.

[18] Sandhu MS, Waterworth DM, Debenham SL, Wheeler E, Papadakis K, Zhao JH (2008). LDL-cholesterol concentrations: a genome-wide association study. Lancet.

[19] Aulchenko YS, Ripatti S, Lindqvist I, Boomsma D, Heid IM, Pramstaller PP (2009). Loci influencing lipid levels and coronary heart disease risk in 16 European population cohorts. Nat Genet.

[20] Kathiresan S, Willer CJ, Peloso GM, Demissie S, Musunuru K, Schadt EE (2009). Common variants at 30 loci contribute to polygenic dyslipidemia. Nat Genet.

[21] Sabatti C, Hartikainen AL, Pouta A, Ripatti S, Brodsky J, Service SK (2009). Genome-wide association analysis of metabolic traits in a birth cohort from a founder population. Nat Genet.

[22] Chasman DI, Pare G, Mora S, Hopewell JC, Peloso G, Clarke R (2009). Forty-three loci associated with plasma lipoprotein size, concentration, and cholesterol content in genome-wide analysis. PLoS Genet.

[23] Teslovich TM, Musunuru K, Smith AV, Edmondson AC, Stylianou IM, Koseki M (2010). Biological, clinical and population relevance of 95 loci for blood lipids. Nature.

[24] Waterworth DM, Ricketts SL, Song K, Chen L, Zhao JH, Ripatti S (2010). Genetic variants influencing circulating lipid levels and risk of coronary artery disease. Arterioscler Thromb Vasc Biol.

[25] Lettre G, Palmer CD, Young T, Ejebe KG, Allayee H, Benjamin EJ (2011). Genome-wide association study of coronary heart disease and its risk factors in 8,090 African Americans: the NHLBI CARe project. PLoS Genet.

[26] Global Lipids Genetics Consortium (2013). Discovery and refinement of loci associated with lipid levels. Nat Genet.

[27] Kathiresan S, Melander O, Anevski D, Guiducci C, Burtt NP, Roos C (2008). Polymorphisms associated with cholesterol and risk of cardiovascular events. N Engl J Med.

[28] Rodrigues AC, Sobrino B, Genvigir FD, Willrich MA, Arazi SS, Dorea EL, et al. Genetic variants in genes related to lipid metabolism and atherosclerosis, dyslipidemia and atorvastatin response. Clin Chim Acta. 2013;417:8-1. 10.1016/j.cca.2012.11.028 23247049

[29] Park MH, Kim N, Lee JY, Park HY (2011). Genetic loci associated with lipid concentrations and cardiovascular risk factors in the Korean population. J Med Genet.

[30] Edmondson AC, Braund PS, Stylianou IM, Khera AV, Nelson CP, Wolfe ML (2011). Dense genotyping of candidate Gene loci identifies variants associated with high-density lipoprotein cholesterol, clinical perspective. Circ Cardiovasc Genet.

[31] Hegele RA, Ban MR, Cao H, AD MI, Robinson JF, Wang J (2015). Targeted next-generation sequencing in monogenic dyslipidemias. Curr Opin Lipidol.

[32] Bolanos-Garcia VM, Miguel RN (2003). On the structure and function of apolipoproteins: more than a family of lipid-binding proteins. Prog Biophys Mol Biol.

[33] Awan Z, Choi HY, Stitziel N, Ruel I, Bamimore MA, Husa R (2013). APOE p. Leu167del mutation in familial hypercholesterolemia. Atherosclerosis.

[34] Marduel M, Ouguerram K, Serre V, Bonnefont-Rousselot D, Marques-Pinheiro A, Erik Berge K, et al. Description of a large family with autosomal dominant hypercholesterolemia associated with the APOE p.Leu167del mutation. Hum Mutat. 2013;34(1):83–87. 10.1002/humu.22215 PMC3638718 22949395

[35] Gautier T, Masson D, Jong MC, Duverneuil L, Le Guern N, Deckert V (2002). Apolipoprotein CI deficiency markedly augments plasma lipoprotein changes mediated by human cholesteryl ester transfer protein (CETP) in CETP transgenic/ApoCI-knocked out mice. J Biol Chem.

[36] Kamboh MI, Aston CE, Hamman RF (2000). DNA sequence variation in human apolipoprotein C4 gene and its effect on plasma lipid profile. Atherosclerosis.

[37] Ridker PM, Paré G, Parker AN, Zee RY, Miletich JP, Chasman DI. Polymorphism in the CETP Gene region, HDL cholesterol, and risk of future myocardial infarction, clinical perspective, 2009. Circ Cardiovasc Genet. 2(1):26–33. 10.1161/CIRCGENETICS.108.817304 PMC2729193 20031564

[38] Lanktree MB, Anand SS, Yusuf S, Hegele RA, SHARE Investigators (2009). Replication of genetic associations with plasma lipoprotein traits in a multiethnic sample. J Lipid Res.

[39] Kettunen J, Tukiainen T, Sarin AP, Ortega-Alonso A, Tikkanen E, Lyytikäinen LP (2012). Genome-wide association study identifies multiple loci influencing human serum metabolite levels. Nat Genet.

[40] Sorci-Thomas MG, Thomas MJ (2002). The effects of altered apolipoprotein AI structure on plasma HDL concentration. Trends Cardiovasc Med.

[41] Arakawa R, Yokoyama S (2002). Helical apolipoproteins stabilize ATP-binding cassette transporter A1 by protecting it from thiol protease-mediated degradation. J Biol Chem.

[42] Stan S, Delvin E, Lambert M, Seidman E, Levy E (2003). Apo A-IV: an update on regulation and physiologic functions. Biochim Biophys Acta Mol Cell Biol Lipids.

[43] Lai CQ, Demissie S, Cupples LA, Zhu Y, Adiconis X, Parnell LD (2004). Influence of the APOA5 locus on plasma triglyceride, lipoprotein subclasses, and CVD risk in the Framingham heart study. J Lipid Res.

[44] Lai CQ, Tai ES, Tan CE, Cutter J, Chew SK, Zhu YP (2003). The APOA5 locus is a strong determinant of plasma triglyceride concentrations across ethnic groups in Singapore. J Lipid Res.

[45] Calandra S, Oliva CP, Tarugi P, Bertolini S (2006). APOA5 and triglyceride metabolism, lesson from human APOA5 deficiency. Curr Opin Lipidol.

[46] Kamatani Y, Matsuda K, Okada Y, Kubo M, Hosono N, Daigo Y, et al. Genome-wide association study of hematological and biochemical traits in a Japanese population, 2010. Nat Genet. 42(3):210–5. 10.1038/ng.531 20139978

[47] Coram MA, Duan Q, Hoffmann TJ, Thornton T, Knowles JW, Johnson NA (2013). Genome-wide characterization of shared and distinct genetic components that influence blood lipid levels in ethnically diverse human populations. Am J Hum Genet.

[48] Tailleux A, Duriez P, Fruchart JC, Clavey V. Apolipoprotein A-II, HDL metabolism and atherosclerosis. Atherosclerosis. 2002;164(1):1–13. 10.1016/s0021-9150(01)00751-1 12119188

[49] Julve J, Rotllan N, Fiévet C, Vallez E, de la Torre C, Ribas V (2010). Human apolipoprotein A-II determines plasma triglycerides by regulating lipoprotein lipase activity and high-density lipoprotein proteome. Arterioscler Thromb Vasc Biol.

[50] Bandarian F, Daneshpour MS, Hedayati M, Naseri M, Azizi F (2016). Identification of sequence variation in the Apolipoprotein A2 Gene and Their relationship with serum high-density lipoprotein cholesterol levels. Iran Biomed J.

[51] Chen SN, Cilingiroglu M, Todd J, Lombardi R, Willerson JT, Gotto AM (2009). Candidate genetic analysis of plasma high-density lipoprotein-cholesterol and severity of coronary atherosclerosis. BMC Med Genet.

[52] Peloso GM, Demissie S, Collins D, Mirel DB, Gabriel SB, Cupples LA (2010). Common genetic variation in multiple metabolic pathways influences susceptibility to low HDL-cholesterol and coronary heart disease. J Lipid Res.

[53] Nakaya Y, Schaefer EJ, Brewer HB. Activation of human post heparin lipoprotein lipase by apolipoprotein H (β2-glycoprotein I). Biochem Biophys Res Commun. 1980;95(3):–1168, 72. 10.1016/0006-291x(80)91595-8 7417307

[54] Lin KY, Pan JP, Yang DL, Huang KT, Chang MS, Ding PY (2001). Evidence for inhibition of low density lipoprotein oxidation and cholesterol accumulation by apolipoprotein H (β2-glycoprotein I). Life Sci.

[55] Asselbergs FW, Guo Y, Van Iperen EP, Sivapalaratnam S, Tragante V, Lanktree MB (2012). Large-scale gene-centric meta-analysis across 32 studies identifies multiple lipid loci. Am J Hum Genet.

[56] Guo T, Yin RX, Li H, Wang YM, Wu JZ, Yang DZ (2015). Association of the Trp316Ser variant (rs1801690) near the apolipoprotein H (β2-glycoprotein-I) gene and serum lipid levels. Int J Clin Exp Pathol.

[57] Borup A, Christensen PM, Nielsen LB, Christoffersen C (2015). Apolipoprotein M in lipid metabolism and cardiometabolic diseases. Curr Opin Lipidol.

[58] Aung LH, Yin RX, Wu DF, Yan TT, Li Q, Wu JZ, et al. Association of the apolipoprotein M gene polymorphisms and serum lipid levels, 2013. Mol Biol Rep. 40(2):1843–53. 10.1007/s11033-012-2240-5 23086303

[59] Zhang Z, Chu G, Yin RX (2013). Apolipoprotein M T-778C polymorphism is associated with serum lipid levels and the risk of coronary artery disease in the Chinese population: a meta-analysis. Lipids Health Dis.

[60] Cao B, Ye YZ, Rui J, Li MQ, Wang W, Wei LY (2013). A single-nucleotide polymorphism in the proximal promoter region of the apolipoprotein M gene is associated with dyslipidaemia but not increased coronary artery diseases in Chinese populations. Lipids Health Dis.

[61] Malaguarnera M, Vacante M, Russo C, Malaguarnera G, Antic T, Malaguarnera L (2012). Lipoprotein (a) in cardiovascular diseases. Biomed Res Int.

[62] Hobbs HH, Brown MS, Goldstein JL (1992). Molecular genetics of the LDL receptor gene in familial hypercholesterolemia. Hum Mutat.

[63] ClinVar (TM). National Center for Biotechnology Information, National Library of Medicine (Bethesda, MD). Available from http://www.ncbi.nlm.nih.gov/clinvar/. Accessed 12 Jan 2017.

[64] Usifo E, Leigh SE, Whittall RA, Lench N, Taylor A, Yeats C (2012). Low-density lipoprotein receptor gene familial hypercholesterolemia variant database: update and pathological assessment. Ann Hum Genet.

[65] Keebler ME, Sanders CL, Surti A, Guiducci C, Burtt NP, Kathiresan S. Association of Blood Lipids with Common DNA sequence variants at 19 genetic loci in the multiethnic United States National Health and nutrition examination survey III, clinical perspective. Circ Cardiovasc Genet. 2009;(2, 3):238–43. 10.1161/CIRCGENETICS.108.829473 PMC3561731 20031591

[66] Fellin R, Arca M, Zuliani G, Calandra S, Bertolini S (2015). The history of autosomal recessive hypercholesterolemia (ARH). From clinical observations to gene identification. Gene.

[67] Beisiegel U, Weber W, Ihrke G, Herz J, Stanley KK (1989). The LDL-receptor-related protein, LRP, is an apolipoprotein E-binding protein. Nature.

[68] de Gonzalo-Calvo D, Cenarro A, Martínez-Bujidos M, Badimon L, Bayes-Genis A, Ordonez-Llanos J (2015). Circulating soluble low-density lipoprotein receptor-related protein 1 (sLRP1) concentration is associated with hypercholesterolemia: a new potential biomarker for atherosclerosis. Int J Cardiol.

[69] Aledo R, Alonso R, Mata P, Llorente-Cortés V, Padró T, Badimon L (2012). LRP1 gene polymorphisms are associated with premature risk of cardiovascular disease in patients with familial hypercholesterolemia. Rev Esp Cardiol (English ed).

[70] Kounnas MZ, Chappell DA, Strickland DK, Argraves WS (1993). Glycoprotein 330, a member of the low density lipoprotein receptor family, binds lipoprotein lipase in vitro. J Biol Chem.

[71] Nykjaer A, Nielsen M, Lookene A, Meyer N, Røigaard H, Etzerodt M (1994). A carboxyl-terminal fragment of lipoprotein lipase binds to the low density lipoprotein receptor-related protein and inhibits lipase-mediated uptake of lipoprotein in cells. J Biol Chem.

[72] Kounnas MZ, Argraves WS, Strickland DK (1992). The 39-kDa receptor-associated protein interacts with two members of the low density lipoprotein receptor family, alpha 2-macroglobulin receptor and glycoprotein 330. J Biol Chem.

[73] González P, Alvarez R, Reguero JR, Batalla A, Alvarez V, Cortina A (2002). Variation in the lipoprotein receptor-related protein, alpha2-macroglobulin and lipoprotein receptor-associated protein genes in relation to plasma lipid levels and risk of early myocardial infarction. Coron Artery Dis.

[74] Argraves KM, Battey FD, CD MC, KR MC, Gåfvels M, Kozarsky KF (1995). The very low density lipoprotein receptor mediates the cellular catabolism of lipoprotein lipase and urokinase-plasminogen activator inhibitor type I complexes. J Biol Chem.

[75] Chen X, Guo Z, Okoro EU, Zhang H, Zhou L, Lin X (2012). Up-regulation of ATP binding cassette transporter A1 expression by very low density lipoprotein receptor and apolipoprotein E receptor 2. J Biol Chem.

[76] Rhainds D, Brissette L (2004). The role of scavenger receptor class B type I (SR-BI) in lipid trafficking: defining the rules for lipid traders. Int J Biochem Cell Biol.

[77] Yang XP, Amar MJ, Vaisman B, Bocharov AV, Vishnyakova TG, Freeman LA (2013). Scavenger receptor-BI is a receptor for lipoprotein (a). J Lipid Res.

[78] Armstrong SM, Sugiyama MG, Fung KY, Gao Y, Wang C, Levy AS (2015). A novel assay uncovers an unexpected role for SR-BI in LDL transcytosis. Cardiovasc Res.

[79] van Bennekum A, Werder M, Thuahnai ST, Han CH, Duong P, Williams DL (2005). Class B scavenger receptor-mediated intestinal absorption of dietary β-carotene and cholesterol. Biochemistry.

[80] Roberts CG, Shen H, Mitchell BD, Damcott CM, Shuldiner AR, Rodriguez A (2007). Variants in scavenger receptor class B type I gene are associated with HDL cholesterol levels in younger women. Hum Hered.

[81] Wu DF, Yin RX, Hu XJ, Aung LH, Cao XL, Miao L (2012). Association of rs5888 SNP in the scavenger receptor class B type 1 gene and serum lipid levels. Lipids Health Dis.

[82] Wu DF, Yin RX, Cao XL, Chen WX, Aung LH, Wang W (2013). Scavenger receptor class B type 1 gene rs5888 single nucleotide polymorphism and the risk of coronary artery disease and ischemic stroke: a case–control study. Int J Med Sci.

[83] Stanislovaitiene D, Lesauskaite V, Zaliuniene D, Smalinskiene A, Gustiene O, Zaliaduonyte-Peksiene D (2013). SCARB1 single nucleotide polymorphism (rs5888) is associated with serum lipid profile and myocardial infarction in an age-and gender-dependent manner. Lipids Health Dis.

[84] Niemsiri V, Wang X, Pirim D, Radwan ZH, Hokanson JE, Hamman RF (2014). Impact of genetic variants in human scavenger receptor class B type I (SCARB1) on plasma lipid traits. Circ Cardiovasc Genet.

[85] Niemsiri V, Wang X, Pirim D, Radwan ZH, Bunker CH, Barmada MM (2015). Genetic contribution of SCARB1 variants to lipid traits in African blacks: a candidate gene association study. BMC Med Genet.

[86] Mead JR, Irvine SA, Ramji DP (2002). Lipoprotein lipase: structure, function, regulation, and role in disease. J Mol Med.

[87] Bruin TD, Mailly F, Barlingen HV, Fisher R, Cabezas MC, Talmud P (1996). Lipoprotein lipase gene mutations D9N and N291S in four pedigrees with familial combined hyperlipidaemia. Eur J Clin Investig.

[88] Wittekoek ME, Pimstone SN, Reymer PW, Feuth L, Botma GJ, Defesche JC (1998). A common mutation in the lipoprotein lipase gene (N291S) alters the lipoprotein phenotype and risk for cardiovascular disease in patients with familial hypercholesterolemia. Circulation.

[89] Wittekoek ME, Moll E, Pimstone SN, Trip MD, Lansberg PJ, Defesche JC (1999). A frequent mutation in the lipoprotein lipase gene (D9N) deteriorates the biochemical and clinical phenotype of familial hypercholesterolemia. Arterioscler Thromb Vasc Biol.

[90] Kolářová H, Tesařová M, Švecová Š, Stránecký V, Přistoupilová A, Zima T (2014). Lipoprotein lipase deficiency: clinical, biochemical and molecular characteristics in three patients with novel mutations in the LPL gene. Folia Biol.

[91] Heid IM, Boes E, Müller M, Kollerits B, Lamina C, Coassin S (2008). Genome-wide association analysis of high-density lipoprotein cholesterol in the population-based KORA study sheds new light on Intergenic regions. Clinical perspective. Circ Cardiovasc Genet.

[92] Ma L, Yang J, Runesha HB, Tanaka T, Ferrucci L, Bandinelli S (2010). Genome-wide association analysis of total cholesterol and high-density lipoprotein cholesterol levels using the Framingham heart study data. BMC Med Genet.

[93] Zabaneh D, Balding DJ (2010). A genome-wide association study of the metabolic syndrome in Indian Asian men. PLoS One.

[94] Zhou L, He M, Mo Z, Wu C, Yang H, Yu D (2013). A genome wide association study identifies common variants associated with lipid levels in the Chinese population. PLoS One.

[95] Rousset X, Vaisman B, Amar M, Sethi AA, Remaley AT (2009). Lecithin: cholesterol acyltransferase–from biochemistry to role in cardiovascular disease. Curr Opin Endocrinol Diabetes Obes.

[96] Cohen JC, Kiss RS, Pertsemlidis A, Marcel YL, McPherson R, Hobbs HH (2004). Multiple rare alleles contribute to low plasma levels of HDL cholesterol. Science.

[97] Holleboom AG, Kuivenhoven JA, Peelman F, Schimmel AW, Peter J, Defesche JC (2011). High prevalence of mutations in LCAT in patients with low HDL cholesterol levels in The Netherlands: identification and characterization of eight novel mutations. Hum Mutat.

[98] Naseri M, Hedayati M, Daneshpour MS, Bandarian F, Azizi F (2014). Identification of genetic variants of lecithin cholesterol acyltransferase in individuals with high HDL-C levels. Mol Med Rep.

[99] Chang TY, Chang CC, Lin S, Yu C, Li BL, Miyazaki A (2001). Roles of acyl-coenzyme a: cholesterol acyltransferase-1 and-2. Curr Opin Lipidol.

[100] Klos KL, Sing CF, Boerwinkle E, Hamon SC, Rea TJ, Clark A (2006). Consistent effects of genes involved in reverse cholesterol transport on plasma lipid and apolipoprotein levels in CARDIA participants. Arterioscler Thromb Vasc Biol.

[101] Wu DF, Yin RX, Aung LH, Hu XJ, Cao XL, Miao L (2010). Polymorphism of rs1044925 in the acyl-CoA: cholesterol acyltransferase-1 gene and serum lipid levels in the Guangxi Bai Ku Yao and Han populations. Lipids Health Dis.

[102] Santamarina-Fojo S, González-Navarro H, Freeman L, Wagner E, Nong Z (2004). Hepatic lipase, lipoprotein metabolism, and atherogenesis. Arterioscler Thromb Vasc Biol.

[103] Lindi V, Schwab U, Louheranta A, Vessby B, Hermansen K, Tapsell L (2008). The G-250A polymorphism in the hepatic lipase gene promoter is associated with changes in hepatic lipase activity and LDL cholesterol: the KANWU study. Nutr Metab Cardiovasc Dis.

[104] Johannsen TH, Kamstrup PR, Andersen RV, Jensen GB, Sillesen H, Tybjærg-Hansen A, et al. Hepatic lipase, genetically elevated high-density lipoprotein, and risk of ischemic cardiovascular disease. J Clin Endocrinol Metab. 2009;94(4):1264–73. 10.1210/jc.2008-1342 19088157

[105] Fan YM, Raitakari OT, Kähönen M, Hutri-Kähönen N, Juonala M, Marniemi J (2009). Hepatic lipase promoter C-480T polymorphism is associated with serum lipids levels, but not subclinical atherosclerosis: the cardiovascular risk in young Finns study. Clin Genet.

[106] Hodoğlugil U, Williamson DW, Mahley RW (2010). Polymorphisms in the hepatic lipase gene affect plasma HDL-cholesterol levels in a Turkish population. J Lipid Res.

[107] Meng L, Ruixing Y, Yiyang L, Xingjiang L, Kela L, Wanying L (2010). Association of LIPC-250G> a polymorphism and several environmental factors with serum lipid levels in the Guangxi Bai Ku Yao and Han populations. Lipids Health Dis.

[108] Weissglas-Volkov D, Aguilar-Salinas CA, Nikkola E, Deere KA, Cruz-Bautista I, Arellano-Campos O, et al. Genomic study in Mexicans identifies a new locus for triglycerides and refines European lipid loci. J Med Genet. 2013;50(5):298–308. 10.1136/jmedgenet-2012-101461 PMC3917605 23505323

[109] Strauss JG, Zimmermann R, Hrzenjak A, Yonggang ZH, Kratky D, Levak-Frank S (2002). Endothelial cell-derived lipase mediates uptake and binding of high-density lipoprotein (HDL) particles and the selective uptake of HDL-associated cholesterol esters independent of its enzymic activity. Biochem J.

[110] Cohen JC (2003). Endothelial lipase: direct evidence for a role in HDL metabolism. J Clin Invest.

[111] Broedl UC, Maugeais C, Millar JS, Jin W, Moore RE, Fuki IV, et al. Endothelial lipase promotes the catabolism of ApoB-containing lipoproteins. Circ Res. 2004;94(12):1554–61. 10.1161/01.RES.0000130657.00222.39 15117821

[112] Singaraja RR, Sivapalaratnam S, Hovingh K, Dubé MP, Castro-Perez J, Collins HL (2013). The impact of partial and complete loss-of-function mutations in endothelial lipase on high-density lipoprotein levels and functionality in humans, clinical perspective. Circ Cardiovasc Genet.

[113] Brown RJ, Edmondson AC, Griffon N, Hill TB, Fuki IV, Badellino KO (2009). A naturally occurring variant of endothelial lipase associated with elevated HDL exhibits impaired synthesis. J Lipid Res.

[114] Liu WY, Yin RX, Zhang L, Cao XL, Miao L, Wu DF (2010). Association of the LIPG 584C> T polymorphism and serum lipid levels in the Guangxi Bai Ku Yao and Han populations. Lipids Health Dis.

[115] Cai G, Huang Z, Zhang B, Weng W, Shi G (2014). The associations between endothelial lipase 584C/T polymorphism and HDL-C level and coronary heart disease susceptibility: a meta-analysis. Lipids Health Dis.

[116] Wang F, Wang W, Wähälä K, Adlercreutz H, Ikonen E, Tikkanen MJ (2008). Role of lysosomal acid lipase in the intracellular metabolism of LDL-transported dehydroepiandrosterone-fatty acyl esters. Am J Physiol Endocrinol Metab.

[117] Fouchier SW, Defesche JC (2013). Lysosomal acid lipase a and the hypercholesterolaemic phenotype. Curr Opin Lipidol.

[118] Muntoni S, Wiebusch H, Jansen-Rust M, Rust S, Schulte H, Berger K (2013). Heterozygosity for lysosomal acid lipase E8SJM mutation and serum lipid concentrations. Nutr Metab Cardiovasc Dis.

[119] Vargas-Alarcón G, Posadas-Romero C, Villarreal-Molina T, Alvarez-León E, Angeles J, Vallejo M (2013). Single nucleotide polymorphisms within LIPA (Lysosomal acid lipase a) gene are associated with susceptibility to premature coronary artery disease. A replication in the genetic of atherosclerotic disease (GEA) Mexican study. PLoS One.

[120] Buhaescu I, Izzedine H (2007). Mevalonate pathway: a review of clinical and therapeutical implications. Clin Biochem.

[121] Akadam-Teker B, Kurnaz O, Coskunpinar E, Daglar-Aday A, Kucukhuseyin O, Cakmak HA (2013). The effects of age and gender on the relationship between HMGCR promoter-911 SNP (rs33761740) and serum lipids in patients with coronary heart disease. Gene.

[122] Bhanushali AA, Contractor A, Das BR (2012). Transl res. Evaluation of the promoter polymorphism -911C>a in the 3-hydroxy-3-methylglutaryl-coenzyme a reductase gene with coronary artery disease risk and cholesterol levels in a population from Western India. Transl Res.

[123] Burkhardt R, Kenny EE, Lowe JK, Birkeland A, Josowitz R, Noel M (2008). Common SNPs in HMGCR in micronesians and whites associated with LDL-cholesterol levels affect alternative splicing of exon13. Arterioscler Thromb Vasc Biol.

[124] Fogarty MP, Xiao R, Prokunina-Olsson L, Scott LJ, Mohlke KL (2010). Allelic expression imbalance at high-density lipoprotein cholesterol locus MMAB-MVK. Hum Mol Genet.

[125] Russell DW (2003). The enzymes, regulation, and genetics of bile acid synthesis. Annu Rev Biochem.

[126] Pullinger CR, Eng C, Salen G, Shefer S, Batta AK, Erickson SK (2002). Human cholesterol 7α-hydroxylase (CYP7A1) deficiency has a hypercholesterolemic phenotype. J Clin Invest.

[127] Liao YC, Lin HF, Rundek T, Cheng R, Hsi E, Sacco RL (2008). Multiple genetic determinants of plasma lipid levels in Caribbean Hispanics. Clin Biochem.

[128] Cai Q, Wang ZQ, Cai Q, Li C, Chen EZ, Jiang ZY (2014). Relationship between CYP7A1-204A> C polymorphism with gallbladder stone disease and serum lipid levels: a meta-analysis. Lipids Health Dis.

[129] Li Q, Hong J, Wu J, Huang ZX, Li QJ, Yin RX (2014). The role of common variants of ABCB1 and CYP7A1 genes in serum lipid levels and lipid-lowering efficacy of statin treatment: a meta-analysis. J Clin Lipidol.

[130] Glaser C, Heinrich J, Koletzko B (2010). Role of FADS1 and FADS2 polymorphisms in polyunsaturated fatty acid metabolism. Metabolism.

[131] Nakayama K, Bayasgalan T, Tazoe F, Yanagisawa Y, Gotoh T, Yamanaka K (2010). A single nucleotide polymorphism in the FADS1/FADS2 gene is associated with plasma lipid profiles in two genetically similar Asian ethnic groups with distinctive differences in lifestyle. Hum Genet.

[132] Kwak JH, Paik JK, Kim OY, Jang Y, Lee SH, Ordovas JM (2011). FADS gene polymorphisms in Koreans: association with ω6 polyunsaturated fatty acids in serum phospholipids, lipid peroxides, and coronary artery disease. Atherosclerosis.

[133] Standl M, Lattka E, Stach B, Koletzko S, Bauer CP, von Berg A (2012). FADS1 FADS2 gene cluster, PUFA intake and blood lipids in children: results from the GINIplus and LISAplus studies. PLoS One.

[134] Lange LA, Hu Y, Zhang H, Xue C, Schmidt EM, Tang ZZ (2014). Whole-exome sequencing identifies rare and low-frequency coding variants associated with LDL cholesterol. Am J Hum Genet.

[135] Yang LI, Qian GA, Zhang X, Huang L, Kui XU, Hu YQ (2017). PNPLA5-knockout rats induced by CRISPR/Cas9 exhibit abnormal bleeding and lipid level. J Integr Agr.

[136] Dean M, Hamon Y, Chimini G (2001). The human ATP-binding cassette (ABC) transporter superfamily. J Lipid Res.

[137] Jones PM, George AM (2004). The ABC transporter structure and mechanism: perspectives on recent research. Cell Mol Life Sci.

[138] Lee MH, Lu K, Hazard S, Yu H, Shulenin S, Hidaka H (2001). Identification of a gene, ABCG5, important in the regulation of dietary cholesterol absorption. Nat Genet.

[139] Small DM (2003). Role of ABC transporters in secretion of cholesterol from liver into bile. Proc Natl Acad Sci.

[140] Garcia-Rios A, Perez-Martinez P, Fuentes F, Mata P, Lopez-Miranda J, Alonso R (2010). Genetic variations at ABCG5/G8 genes modulate plasma lipids concentrations in patients with familial hypercholesterolemia. Atherosclerosis.

[141] Caamano JM, Pacheco A, Lanas F, Salazar LA (2008). Single nucleotide polymorphisms in ABCG5 and ABCG8 genes in Chilean subjects with polygenic hypercholesterolemia and controls. Clin Chem Lab Med.

[142] Chen ZC, Shin SJ, Kuo KK, Lin KD, Yu ML, Hsiao PJ (2008). Significant association of ABCG8: D19H gene polymorphism with hypercholesterolemia and insulin resistance. J Hum Genet.

[143] Rudkowska I, Jones PJ (2008). Polymorphisms in ABCG5/G8 transporters linked to hypercholesterolemia and gallstone disease. Nutr Rev.

[144] Li Q, Wei XL, Yin RX (2012). Association of ATP binding cassette transporter G8 rs4148217 SNP and serum lipid levels in Mulao and Han nationalities. Lipids Health Dis.

[145] Kaminski WE, Piehler A, Wenzel JJ (2006). ABC A-subfamily transporters: structure, function and disease. Biochim Biophys Acta Mol basis Dis.

[146] Yvan-Charvet L, Wang N, Tall AR (2010). Role of HDL, ABCA1, and ABCG1 transporters in cholesterol efflux and immune responses. Arterioscler Thromb Vasc Biol.

[147] Zago VH, Scherrer DZ, Parra ES, Panzoldo NB, Alexandre F, Nakandakare ER (2015). Association between ABCG1 polymorphism rs1893590 and high-density lipoprotein (HDL) in an asymptomatic Brazilian population. Mol Biol Rep.

[148] Thompson R, Strautnieks S (2001). BSEP: function and role in progressive familial intrahepatic cholestasis. Semin Liver Dis.

[149] Andreotti G, Menashe I, Chen J, Chang SC, Rashid A, Gao YT (2009). Genetic determinants of serum lipid levels in Chinese subjects: a population-based study in shanghai, China. Eur J Epidemiol.

[150] Elferink RP, Paulusma CC (2007). Function and pathophysiological importance of ABCB4 (MDR3 P-glycoprotein). Pflugers Arch.

[151] Acalovschi M, Tirziu S, Chiorean E, Krawczyk M, Grünhage F, Lammert F (2009). Common variants of ABCB4 and ABCB11 and plasma lipid levels: a study in sib pairs with gallstones, and controls. Lipids.

[152] Nies AT, Keppler D (2007). The apical conjugate efflux pump ABCC2 (MRP2). Pflugers Arch.

[153] Motazacker MM, Peter J, Treskes M, Shoulders CC, Kuivenhoven JA, Hovingh GK (2013). Evidence of a polygenic origin of extreme high-density lipoprotein cholesterol levels significance. Arterioscler Thromb Vasc Biol.

[154] Pomozi V, Le Saux O, Brampton C, Apana A, Iliás A, Szeri F (2013). ABCC6 is a basolateral plasma membrane protein. Circ Res.

[155] Kuzaj P, Kuhn J, Dabisch-Ruthe M, Faust I, Götting C, Knabbe C (2014). ABCC6-a new player in cellular cholesterol and lipoprotein metabolism?. Lipids Health Dis.

[156] Tsuruoka S, Ishibashi K, Yamamoto H, Wakaumi M, Suzuki M, Schwartz GJ (2002). Functional analysis of ABCA8, a new drug transporter. Biochem Biophys Res Commun.

[157] Jia L, Betters JL, Yu L (2011). Niemann-pick C1-like 1 (NPC1L1) protein in intestinal and hepatic cholesterol transport. Annu Rev Physiol.

[158] Cohen JC, Pertsemlidis A, Fahmi S, Esmail S, Vega GL, Grundy SM, et al. Multiple rare variants in NPC1L1 associated with reduced sterol absorption and plasma low-density lipoprotein levels. Proc Nat Acad Sci USA. 2006;103(6):1810–5. 10.1073/pnas.0508483103 PMC1413637 16449388

[159] Polisecki E, Peter I, Simon JS, Hegele RA, Robertson M, Ford I (2010). Genetic variation at the NPC1L1 gene locus, plasma lipoproteins, and heart disease risk in the elderly. J Lipid Res.

[160] Martín B, Solanas-Barca M, García-Otín ÁL, Pampín S, Cofán M, Ros E (2010). An NPC1L1 gene promoter variant is associated with autosomal dominant hypercholesterolemia. Nutr Metab Cardiovasc Dis.

[161] Miao L, Yin RX, Hu XJ, Wu DF, Cao XL, Li Q (2012). Association of rs2072183 SNP and serum lipid levels in the Mulao and Han populations. Lipids Health Dis.

[162] Barter PJ, Brewer HB, Chapman MJ, Hennekens CH, Rader DJ, Tall AR (2003). Cholesteryl ester transfer protein. Arterioscler Thromb Vasc Biol.

[163] de Grooth GJ, Smilde TJ, van Wissen S, Klerkx AH, Zwinderman AH, Fruchart JC (2004). The relationship between cholesteryl ester transfer protein levels and risk factor profile in patients with familial hypercholesterolemia. Atherosclerosis.

[164] Hogue JC, Lamarche B, Gaudet D, Larivière M, Tremblay AJ, Bergeron J (2004). Relationship between cholesteryl ester transfer protein and LDL heterogeneity in familial hypercholesterolemia. J Lipid Res.

[165] Hiura Y, Shen CS, Kokubo Y, Okamura T, Morisaki T, Tomoike H (2009). Identification of genetic markers associated with high-density lipoprotein-cholesterol by genome-wide screening in a Japanese population. Circulation.

[166] Ko A, Cantor RM, Weissglas-volkov D, Nikkola E, Reddy PM, Sinsheimer JS (2014). Amerindian-specific regions under positive selection harbour new lipid variants in Latinos. Nat Commun.

[167] Albers JJ, Vuletic S, Cheung MC (2012). Role of plasma phospholipid transfer protein in lipid and lipoprotein metabolism. Biochim Biophys Acta Mol Cell Biol Lipids.

[168] Aouizerat BE, Engler MB, Natanzon Y, Kulkarni M, Song J, Eng C (2006). Genetic variation of PLTP modulates lipoprotein profiles in hypoalphalipoproteinemia. J Lipid Res.

[169] Engler MB, Pullinger CR, Malloy MJ, Natanzon Y, Kulkarni MV, Song J (2008). Genetic variation in phospholipid transfer protein modulates lipoprotein profiles in hyperalphalipoproteinemia. Metabolism.

[170] Raychaudhuri S, Prinz WA (2010). The diverse functions of oxysterol-binding proteins. Annu Rev Cell Dev Biol.

[171] Zhang M, Liu P, Dwyer NK, Christenson LK, Fujimoto T, Martinez F (2002). MLN64 mediates mobilization of lysosomal cholesterol to steroidogenic mitochondria. J Biol Chem.

[172] Seidah NG, Mayer G, Zaid A, Rousselet E, Nassoury N, Poirier S (2008). The activation and physiological functions of the proprotein convertases. Int J Biochem Cell Biol.

[173] Horton JD, Cohen JC, Hobbs HH (2009). PCSK9: a convertase that coordinates LDL catabolism. J Lipid Res.

[174] Jin W, Wang X, Millar JS, Quertermous T, Rothblat GH, Glick JM (2007). Hepatic proprotein convertases modulate HDL metabolism. Cell Metab..

[175] Iatan I, Dastani Z, Do R, Weissglas-Volkov D, Ruel I, Lee JC (2009). Genetic variation at the Proprotein Convertase Subtilisin/Kexin type 5 Gene modulates high-density lipoprotein cholesterol levels. Clinical perspective. Circ Cardiovasc Genet.

[176] Brown MS, Goldstein JL (1997). The SREBP pathway: regulation of cholesterol metabolism by proteolysis of a membrane-bound transcription factor. Cell.

[177] Marschner K, Kollmann K, Schweizer M, Braulke T, Pohl S (2011). A key enzyme in the biogenesis of lysosomes is a protease that regulates cholesterol metabolism. Science.

[178] Willnow TE, Kjølby M, Nykjaer A. Sortilins: new players in lipoprotein metabolism. Curr Opin Lipidol. 2011;22(2):79-85. 10.1097/MOL.0b013e3283416f2b 21124217

[179] Gustafsen C, Kjolby M, Nyegaard M, Mattheisen M, Lundhede J, Buttenschøn H (2014). The hypercholesterolemia-risk gene SORT1 facilitates PCSK9 secretion. Cell Metab.

[180] Strong A, Ding Q, Edmondson AC, Millar JS, Sachs KV, Li X (2012). Hepatic sortilin regulates both apolipoprotein B secretion and LDL catabolism. J Clin Invest.

[181] Kleber ME, Renner W, Grammer TB, Linsel-Nitschke P, Boehm BO, Winkelmann BR (2010). Association of the single nucleotide polymorphism rs599839 in the vicinity of the sortilin 1 gene with LDL and triglyceride metabolism, coronary heart disease and myocardial infarction: the Ludwigshafen risk and cardiovascular Health study. Atherosclerosis.

[182] Zhou YJ, Hong SC, Yang Q, Yin RX, Cao XL, Chen WX (2015). Association of variants in CELSR2-PSRC1-SORT1 with risk of serum lipid traits, coronary artery disease and ischemic stroke. Int J Clin Exp Pathol.

[183] Meroufel DN, Mediene-Benchekor S, Lardjam-Hetraf SA, Ouhaïbi-Djellouli H, Boulenouar H, Hamani-Medjaoui I, et al. Associations of common SNPs in the SORT1, GCKR, LPL, APOA1, CETP, LDLR, APOE genes with lipid trait levels in an Algerian population sample. Int J Clin Exp Pathol 2015;8(6):7358. PMC4525970 26261636

[184] Arvind P, Nair J, Jambunathan S, Kakkar VV, Shanker J (2014). CELSR2–PSRC1–SORT1 gene expression and association with coronary artery disease and plasma lipid levels in an Asian Indian cohort. J Cardiol.

[185] Nakayama K, Bayasgalan T, Yamanaka K, Kumada M, Gotoh T, Utsumi N (2009). Large scale replication analysis of loci associated with lipid concentrations in a Japanese population. J Med Genet.

[186] Shirts BH, Hasstedt SJ, Hopkins PN, Hunt SC (2011). Evaluation of the gene–age interactions in HDL cholesterol, LDL cholesterol, and triglyceride levels: the impact of the SORT1 polymorphism on LDL cholesterol levels is age dependent. Atherosclerosis.

[187] Karlos A, Shearer J, Gnatiuk E, Onyewu C, Many G, Hoffman EP (2012). Effect of the SORT1 low-density lipoprotein cholesterol locus is sex-specific in a fit, Canadian young-adult population. Appl Pysiol Nutr Metab.

[188] Musunuru K, Strong A, Frank-Kamenetsky M, Lee NE, Ahfeldt T, Sachs KV (2010). From noncoding variant to phenotype via SORT1 at the 1p13 cholesterol locus. Nature.

[189] Santulli G (2014). Angiopoietin-like proteins: a comprehensive look. Front Endocrinol.

[190] Zhang R (2016). The ANGPTL3-4-8 model, a molecular mechanism for triglyceride trafficking. Open Biol.

[191] Shimamura M, Matsuda M, Yasumo H, Okazaki M, Fujimoto K, Kono K (2007). Angiopoietin-like protein3 regulates plasma HDL cholesterol through suppression of endothelial lipase. Arterioscler Thromb Vasc Biol.

[192] Nakajima K, Kobayashi J, Mabuchi H, Nakano T, Tokita Y, Nagamine T (2010). Association of angiopoietin-like protein 3 with hepatic triglyceride lipase and lipoprotein lipase activities in human plasma. Ann Clin Biochem.

[193] Wang Y, Gusarova V, Banfi S, Gromada J, Cohen JC, Hobbs HH (2015). Inactivation of ANGPTL3 reduces hepatic VLDL-triglyceride secretion. J Lipid Res.

[194] Legry V, Bokor S, Cottel D, Beghin L, Catasta G, Nagy E (2009). Associations between common genetic polymorphisms in angiopoietin-like proteins 3 and 4 and lipid metabolism and adiposity in European adolescents and adults. J Clin Endocrinol Metab.

[195] Hanson RL, Leti F, Tsinajinnie D, Kobes S, Puppala S, Curran JE (2016). The Arg59Trp variant in ANGPTL8 (betatrophin) is associated with total and HDL-cholesterol in American Indians and Mexican Americans and differentially affects cleavage of ANGPTL3. Mol Gen Metab..

[196] Guo T, Yin RX, Wu J, Lin QZ, Shi GY, Shen SW (2015). Association of the angiopoietin-like protein 8 rs2278426 polymorphism and several environmental factors with serum lipid levels. Mol Med Rep.

[197] Romeo S, Pennacchio LA, Fu Y, Boerwinkle E, Tybjaerg-Hansen A, Hobbs HH (2007). Population-based resequencing of ANGPTL4 uncovers variations that reduce triglycerides and increase HDL. Nat Genet.

[198] Folsom AR, Peacock JM, Demerath E, Boerwinkle E (2008). Variation in ANGPTL4 and risk of coronary heart disease: the atherosclerosis risk in communities study. Metabolism.

[199] Nettleton JA, Volcik KA, Hoogeveen RC, Boerwinkle E (2009). Carbohydrate intake modifies associations between ANGPTL4 [E40K] genotype and HDL-cholesterol concentrations in white men from the atherosclerosis risk in communities (ARIC) study. Atherosclerosis.

[200] Namkung J, Koh SB, Kong ID, Choi JW, Yeh BI (2011). Serum levels of angiopoietin-related growth factor are increased in metabolic syndrome. Metabolism.

[201] Oike Y, Akao M, Yasunaga K, Yamauchi T, Morisada T, Ito Y (2005). Angiopoietin-related growth factor antagonizes obesity and insulin resistance. Nat Med.

[202] Katrine TB, Vester-Christensen MB, Bennett EP, Levery SB, Schwientek T, Yin W (2010). O-Glycosylation modulates Proprotein Convertase activation of Angiopoietin-like protein 3. Possible role of polypeptide GalNAc-transferase-2 in regulation of concentrations of plasma lipids. J Biol Chem.

[203] Tai ES, Sim XL, Ong TH, Wong TY, Saw SM, Aung T (2009). Polymorphisms at newly identified lipid-associated loci are associated with blood lipids and cardiovascular disease in an Asian Malay population. J Lipid Res.

[204] Weissglas-Volkov D, Aguilar-Salinas CA, Sinsheimer JS, Riba L, Huertas-Vazquez A, Ordoñez-Sánchez ML (2010). Investigation of variants identified in Caucasian genome-wide association studies for plasma HDL cholesterol and triglycerides levels in Mexican dyslipidemic study samples. Circ Cardiovasc Genet.

[205] Li Q, Yin RX, Yan TT, Miao L, Cao XL, Hu XJ (2011). Association of the GALNT2 gene polymorphisms and several environmental factors with serum lipid levels in the Mulao and Han populations. Lipids Health Dis.

[206] Guo T, Yin RX, Lin WX, Wang W, Huang F, Pan SL (2016). Association of the variants and haplotypes in the DOCK7, PCSK9 and GALNT2 genes and the risk of hyperlipidaemia. J Cell Mol Med.

[207] Scotti E, Calamai M, Goulbourne CN, Zhang L, Hong C, Lin RR (2013). IDOL stimulates clathrin-independent endocytosis and multivesicular body-mediated lysosomal degradation of the low-density lipoprotein receptor. Mol Cell Biol.

[208] Yan TT, Yin RX, Li Q, Huang P, Zeng XN, Huang KK (2012). Association of MYLIP rs3757354 SNP and several environmental factors with serum lipid levels in the Guangxi Bai Ku Yao and Han populations. Lipids Health Dis.

[209] Weissglas-Volkov D, Calkin AC, Tusie-Luna T, Sinsheimer JS, Zelcer N, Riba L (2011). The N342S MYLIP polymorphism is associated with high total cholesterol and increased LDL receptor degradation in humans. J Clin Invest.

[210] Dhyani A, Tibolla G, Baragetti A, Garlaschelli K, Pellegatta F, Grigore L (2015). IDOL N342S variant, atherosclerosis progression and cardiovascular disorders in the Italian general population. PLoS One.

[211] Raimondo A, Rees MG, Gloyn AL (2015). Glucokinase regulatory protein: complexity at the crossroads of triglyceride and glucose metabolism. Curr Opin Lipidol.

[212] Tracz A, Madzio J, Gnys P, Malachowska B, Borowiec M, Wyka K (2014). Genetic variability of GCKR alters lipid profiles in children with monogenic and autoimmune diabetes. Exp Clin Endocrinol Diabetes.

[213] Lee HJ, Jang HB, Kim HJ, Ahn Y, Hong KW, Cho SB (2015). The dietary monounsaturated to saturated fatty acid ratio modulates the genetic effects of GCKR on serum lipid levels in children. Clin Chim Acta.

[214] Zhao Y, Ma Y, Fang Y, Liu L, Wu S, Fu D (2012). Association between PON1 activity and coronary heart disease risk: a meta-analysis based on 43 studies. Mol Gen Metab.

[215] Lahiry P, Ban MR, Pollex RL, Sawyez CG, Huff MW, Young TK (2007). Common variants APOC3, APOA5, APOE and PON1 are associated with variation in plasma lipoprotein traits in Greenlanders. Int J Circumpolar Health.

[216] van Himbergen TM, van Tits LJ, Ter Avest E, Roest M, Voorbij HA, de Graaf J (2008). Paraoxonase (PON1) is associated with familial combined hyperlipidemia. Atherosclerosis.

[217] Pérez-Herrera N, May-Pech C, Hernández-Ochoa I, Castro-Mañé J, Rojas-García E, Borja-Aburto VH (2008). PON1Q192R polymorphism is associated with lipid profile in Mexican men with Mayan ascendancy. Exp Mol Pathol.

[218] Chang MH, Yesupriya A, Ned RM, Mueller PW, Dowling NF (2010). Genetic variants associated with fasting blood lipids in the US population: third National Health and nutrition examination survey. BMC Med Genet.

[219] Scherrer DZ, Zago VH, Vieira IC, Parra ES, Panzoldo NB, Alexandre F (2015). P.Q192R SNP of PON1 seems not to be associated with carotid atherosclerosis risk factors in an asymptomatic and Normolipidemic Brazilian population sample. Arq Bras Cardiol.

[220] Yan TT, Yin RX, Li Q, Huang P, Zeng XN, Huang KK (2011). Sex-specific association of rs16996148 SNP in the NCAN/CILP2/PBX4 and serum lipid levels in the Mulao and Han populations. Lipids Health Dis.

[221] Lin CY, Huang ZH, Mazzone T (2001). Interaction with proteoglycans enhances the sterol efflux produced by endogenous expression of macrophage apoE. J Lipid Res.

[222] Zhang Y, Gan W, Tian C, Li H, Lin X, Chen Y (2013). Association of PPP1R3B polymorphisms with blood lipid and C-reactive protein levels in a Chinese population. J Diabetes.

[223] Shimano H (2001). Sterol regulatory element-binding proteins (SREBPs): transcriptional regulators of lipid synthetic genes. Prog Lipid Res.

[224] Llorente-Cortés V, Costales P, Bernués J, Camino-Lopez S, Badimon L (2006). Sterol regulatory element-binding protein-2 negatively regulates low density lipoprotein receptor-related protein transcription. J Mol Biol.

[225] Wong J, Quinn CM, Brown AJ (2006). SREBP-2 positively regulates transcription of the cholesterol efflux gene, ABCA1, by generating oxysterol ligands for LXR. Biochem J.

[226] Alrefai WA, Annaba F, Sarwar Z, Dwivedi A, Saksena S, Singla A (2007). Modulation of human Niemann-pick C1-like 1 gene expression by sterol: role of sterol regulatory element binding protein 2. Am J Physiol Gastrointest Liver Physiol.

[227] Jeong HJ, Lee HS, Kim KS, Kim YK, Yoon D, Park SW (2008). Sterol-dependent regulation of proprotein convertase subtilisin/kexin type 9 expression by sterol-regulatory element binding protein-2. J Lipid Res.

[228] Kivelä AM, Dijkstra MH, Heinonen SE, Gurzeler E, Jauhiainen S, Levonen AL (2012). Regulation of endothelial lipase and systemic HDL cholesterol levels by SREBPs and VEGF-A. Atherosclerosis.

[229] Laaksonen R, Thelen KM, Päivä H, Matinheikki J, Vesalainen R, Janatuinen T (2006). Genetic variant of the SREBF-1 gene is significantly related to cholesterol synthesis in man. Atherosclerosis.

[230] Védie B, Jeunemaitre X, Mégnien JL, Atger V, Simon A, Moatti N (2001). A new DNA polymorphism in the 5′ untranslated region of the human SREBP-1a is related to development of atherosclerosis in high cardiovascular risk population. Atherosclerosis.

[231] Yaju D, Ruixing Y, Yiyang L, Yijiang Z, Weixiong L, Dezhai Y (2009). Polymorphism of the sterol regulatory element-binding protein-2 gene and its association with serum lipid levels in the Guangxi Hei Yi Zhuang and Han populations. Am J Med Sci.

[232] Liu X, Li Y, Lu X, Wang L, Zhao Q, Yang W (2010). Interactions among genetic variants from SREBP2 activating-related pathway on risk of coronary heart disease in Chinese Han population. Atherosclerosis.

[233] Durst R, Jansen A, Erez G, Bravdo R, Butbul E, Avi LB (2006). The discrete and combined effect of SREBP-2 and SCAP isoforms in the control of plasma lipids among familial hypercholesterolaemia patients. Atherosclerosis.

[234] Goldstein JL, DeBose-Boyd RA, Brown MS (2006). Protein sensors for membrane sterols. Cell.

[235] Kaulfers AM, Deka R, Dolan L, Martin LJ (2015). Association of INSIG2 polymorphism with overweight and LDL in children. PLoS One.

[236] Hubácek JA, Suchánek P, Lánská V, Pitha J, Adámková V (2011). INSIG2 G-102A promoter variant exhibits context--dependent effect on HDL-cholesterol levels but not on BMI in Caucasians. Folia Biol.

[237] Oki K, Yamane K, Kamei N, Asao T, Awaya T, Kohno N (2009). The single nucleotide polymorphism upstream of insulin-induced gene 2 (INSIG2) is associated with the prevalence of hypercholesterolaemia, but not with obesity, in Japanese American women. Br J Nutr.

[238] Do R, Bailey SD, Paré G, Montpetit A, Desbiens K, Hudson TJ (2010). Fine mapping of the insulin-induced Gene 2 identifies a variant associated with LDL cholesterol and Total Apolipoprotein B levels. Clinical perspective. Circ Cardiovasc Genet.

[239] Schrem H, Klempnauer J, Borlak J (2002). Liver-enriched transcription factors in liver function and development. Part I: the hepatocyte nuclear factor network and liver-specific gene expression. Pharmacol Rev.

[240] Pramfalk C, Jiang ZY, Cai Q, Hu H, Zhang SD, Han TQ (2010). HNF1α and SREBP2 are important regulators of NPC1L1 in human liver. J Lipid Res.

[241] Li H, Dong B, Park SW, Lee HS, Chen W, Liu J (2009). Hepatocyte nuclear factor 1α plays a critical role in PCSK9 gene transcription and regulation by the natural hypocholesterolemic compound berberine. J Biol Chem.

[242] Nair AK, Piaggi P, McLean NA, Kaur M, Kobes S, Knowler WC (2016). Assessment of established HDL-C loci for association with HDL-C levels and type 2 diabetes in pima Indians. Diabetologia.

[243] Zhao C, Dahlman-Wright K (2010). Liver X receptor in cholesterol metabolism. J Endocrinol.

[244] Burkhardt R, Toh SA, Lagor WR, Birkeland A, Levin M, Li X (2010). Trib1 is a lipid-and myocardial infarction–associated gene that regulates hepatic lipogenesis and VLDL production in mice. J Clin Invest.

[245] Bauer RC, Yenilmez BO, Rader DJ (2015). Tribbles-1: a novel regulator of hepatic lipid metabolism in humans. Biochem Soc Trans.

[246] Varbo A, Benn M, Tybjærg-Hansen A, Grande P, Nordestgaard BG (2011). TRIB1 and GCKR polymorphisms, lipid levels, and risk of ischemic heart disease in the general population. Arterioscler Thromb Vasc Biol.

[247] Aung LH, Yin RX, Wu DF, Li Q, Yan TT, Wang YM (2011). Association of the TRIB1 tribbles homolog 1 gene rs17321515 a> G polymorphism and serum lipid levels in the Mulao and Han populations. Lipids Health Dis.

[248] Duval C, Müller M, Kersten S (2007). PPARα and dyslipidemia. Biochim Biophys Acta Mol Cell Biol Lipids.

[249] Semple RK, Chatterjee VK, O’Rahilly S (2006). PPARγ and human metabolic disease. J Clin Invest.

[250] Seedorf U, Aberle J (2007). Emerging roles of PPARδ in metabolism. Biochim Biophys Acta Mol Cell Biol Lipids.

[251] Chen ES, Mazzotti DR, Furuya TK, Cendoroglo MS, Ramos LR, Araujo LQ (2010). Association of PPARα gene polymorphisms and lipid serum levels in a Brazilian elderly population. Exp Mol Pathol.

[252] Gu SJ, Guo ZR, Zhou ZY, Hu XS, Wu M (2014). PPAR α and PPAR γ polymorphisms as risk factors for Dyslipidemia in a Chinese Han population. Lipids Health Dis.

[253] Fan W, Shen C, Wu M, Zhou ZY, Guo ZR (2015). Association and interaction of PPARα, δ, and γ Gene polymorphisms with low-density lipoprotein-cholesterol in a Chinese Han population. Genet Test Mol Biomarkers.

[254] Chawla A, Repa JJ, Evans RM, Mangelsdorf DJ (2001). Nuclear receptors and lipid physiology: opening the X-files. Science.

[255] Stoeckman AK, Ma L, Towle HC (2004). Mlx is the functional heteromeric partner of the carbohydrate response element-binding protein in glucose regulation of lipogenic enzyme genes. J Biol Chem.

[256] Aken BL, Ayling S, Barrell D, Clarke L, Curwen V, Fairley S, et al. The Ensembl gene annotation system. Database. 2016; doi:10.1093/database/baw093. 10.1093/database/baw093 PMC4919035 27337980

[257] O'Leary NA, Wright MW, Brister JR, Ciufo S, Haddad D, McVeigh R (2016). Reference sequence (RefSeq) database at NCBI: current status, taxonomic expansion, and functional annotation. Nucleic Acids Res.

[258] Dayem Ullah AZ, Lemoine NR, Chelala C (2013). A practical guide for the functional annotation of genetic variations using SNPnexus. Brief Bioinform.

